# The IG-DMR and the *MEG3*-DMR at Human Chromosome 14q32.2: Hierarchical Interaction and Distinct Functional Properties as Imprinting Control Centers

**DOI:** 10.1371/journal.pgen.1000992

**Published:** 2010-06-17

**Authors:** Masayo Kagami, Maureen J. O'Sullivan, Andrew J. Green, Yoshiyuki Watabe, Osamu Arisaka, Nobuhide Masawa, Kentarou Matsuoka, Maki Fukami, Keiko Matsubara, Fumiko Kato, Anne C. Ferguson-Smith, Tsutomu Ogata

**Affiliations:** 1 Department of Endocrinology and Metabolism, National Research Institute for Child Health and Development, Tokyo, Japan; 2 Department of Pathology, School of Medicine, Our Lady's Children's Hospital, Trinity College, Dublin, Ireland; 3 National Center for Medical Genetics, University College Dublin, Our Lady's Hospital, Dublin, Ireland; 4 School of Medicine and Medical Science, University College, Dublin, Ireland; 5 Department of Pediatrics, Dokkyo University School of Medicine, Tochigi, Japan; 6 Department of Pathology, Dokkyo University School of Medicine, Tochigi, Japan; 7 Department of Pathology, National Center for Child Health and Development, Tokyo, Japan; 8 Department of Physiology, Development and Neuroscience, University of Cambridge, Cambridge, United Kingdom; The Babraham Institute, United Kingdom

## Abstract

Human chromosome 14q32.2 harbors the germline-derived primary *DLK1*-*MEG3* intergenic differentially methylated region (IG-DMR) and the postfertilization-derived secondary *MEG3*-DMR, together with multiple imprinted genes. Although previous studies in cases with microdeletions and epimutations affecting both DMRs and paternal/maternal uniparental disomy 14-like phenotypes argue for a critical regulatory function of the two DMRs for the 14q32.2 imprinted region, the precise role of the individual DMR remains to be clarified. We studied an infant with upd(14)pat body and placental phenotypes and a heterozygous microdeletion involving the IG-DMR alone (patient 1) and a neonate with upd(14)pat body, but no placental phenotype and a heterozygous microdeletion involving the *MEG3*-DMR alone (patient 2). The results generated from the analysis of these two patients imply that the IG-DMR and the *MEG3*-DMR function as imprinting control centers in the placenta and the body, respectively, with a hierarchical interaction for the methylation pattern in the body governed by the IG-DMR. To our knowledge, this is the first study demonstrating an essential long-range imprinting regulatory function for the secondary DMR.

## Author Summary

Genomic imprinting is a process causing genes to be expressed in a parent-of-origin specific manner—some imprinted genes are expressed from maternally inherited chromosomes and others from paternally inherited chromosomes. Imprinted genes are often located in clusters regulated by regions that are differentially methylated according to their parental origin. The human chromosome 14q32.2 imprinted region harbors the germline-derived primary *DLK1*-*MEG3* intergenic differentially methylated region (IG-DMR) and the postfertilization-derived secondary *MEG3*-DMR, together with multiple imprinted genes. Perturbed dosage of these imprinted genes, for example in patients with paternal and maternal uniparental disomy 14, causes distinct phenotypes. Here, through analysis of patients with microdeletions recapitulating some or all of the uniparental disomy 14 phenotypes, we show that the IG-DMR acts as an upstream regulator for the methylation pattern of the *MEG3*-DMR in the body but not in the placenta. Importantly, in the body, the *MEG3*-DMR functions as an imprinting control center. To our knowledge, this is the first study demonstrating an essential function for the secondary DMR in the regulation of multiple imprinted genes. Thus, the results provide a significant advance in the clarification of underlying epigenetic features that can act to regulate imprinting.

## Introduction

Human chromosome 14q32.2 carries a cluster of protein-coding paternally expressed genes (*PEGs*) such as *DLK1* and *RTL1* and non-coding maternally expressed genes (*MEGs*) such as *MEG3* (alias, *GTL2*), *RTL1as* (*RTL1* antisense), *MEG8*, *snoRNAs*, and *microRNAs* [1], [2]. Consistent with this, paternal uniparental disomy 14 (upd(14)pat) results in a unique phenotype characterized by facial abnormality, small bell-shaped thorax, abdominal wall defects, placentomegaly, and polyhydramnios [2], [3], and maternal uniparental disomy 14 (upd(14)mat) leads to less-characteristic but clinically discernible features including growth failure [2], [4].

The 14q32.2 imprinted region also harbors two differentially methylated regions (DMRs), i.e., the germline-derived primary *DLK1*-*MEG3* intergenic DMR (IG-DMR) and the postfertilization-derived secondary *MEG3*-DMR [1], [2]. Both DMRs are hypermethylated after paternal transmission and hypomethylated after maternal transmission in the body, whereas in the placenta the IG-DMR alone remains as a DMR and the *MEG3*-DMR is rather hypomethylated [1], [2]. Furthermore, previous studies in cases with upd(14)pat/mat-like phenotypes have revealed that epimutations (hypermethylation) and microdeletions affecting both DMRs of maternal origin cause paternalization of the 14q32.2 imprinted region, and that epimutations (hypomethylation) affecting both DMRs of paternal origin cause maternalization of the 14q32.2 imprinted region, while microdeletions involving the DMRs of paternal origin have no effect on the imprinting status [2], [5]–[8]. These findings, together with the notion that parent-of-origin specific expression patterns of imprinted genes are primarily dependent on the methylation status of the DMRs [9], argue for a critical regulatory function of the two DMRs for the 14q32.2 imprinted region, with possible different effects between the body and the placenta.

However, the precise role of individual DMR remains to be clarified. Here, we report that the IG-DMR and the *MEG3*-DMR show a hierarchical interaction for the methylation pattern in the body, and function as imprinting control centers in the placenta and the body, respectively. To our knowledge, this is the first study demonstrating not only different roles between the primary and secondary DMRs at a single imprinted region, but also an essential regulatory function for the secondary DMR.

## Results

### Clinical reports

**Figure 1:**
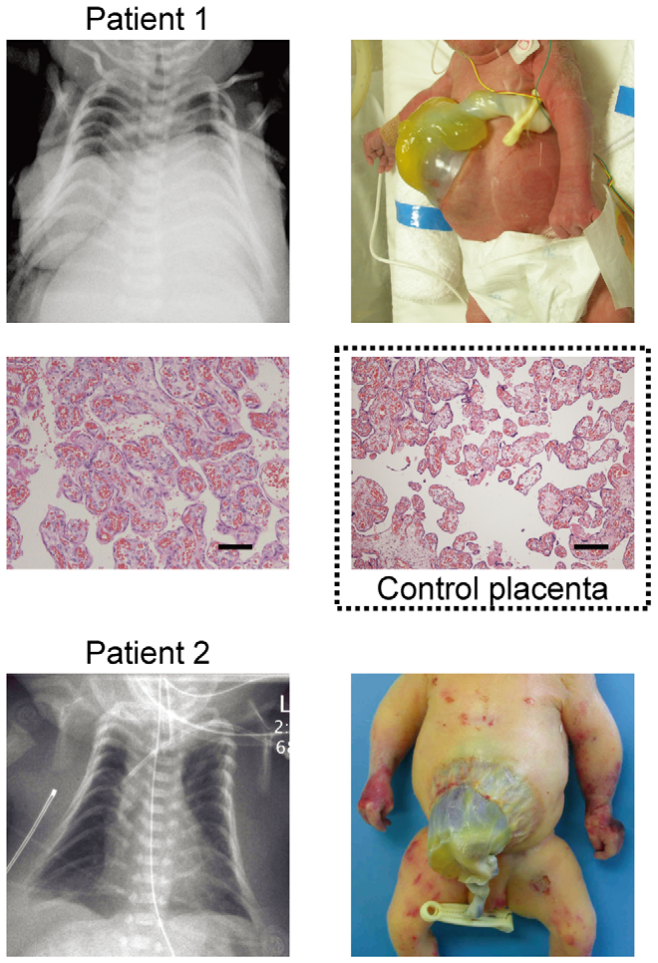
Clinical phenotypes of patients 1 and 2 at birth. Both patients have bell shaped thorax with coat hanger appearance of the ribs and omphalocele. In patient 1, histological examination of the placenta shows proliferation of dilated and congested chorionic villi, as has previously been observed in a case with upd(14)pat [2]. For comparison, the histological finding of a gestational age matched (33 weeks) control placenta is shown in a dashed square. The horizontal black bars indicate 100 µm.

We studied an infant with upd(14)pat body and placental phenotypes (patient 1) and a neonate with upd(14)pat body, but no placental, phenotype (patient 2) (Figure 1). Detailed clinical features of patients 1 and 2 are shown in Table 1. In brief, patient 1 was delivered by a caesarean section at 33 weeks of gestation due to progressive polyhydramnios despite amnioreduction at 28 and 30 weeks of gestation, whereas patient 2 was born at 28 weeks of gestation by a vaginal delivery due to progressive labor without discernible polyhydramnios. Placentomegaly was observed in patient 1 but not in patient 2. Patients 1 and 2 were found to have characteristic face, small bell-shaped thorax with coat hanger appearance of the ribs, and omphalocele. Patient 1 received surgical treatment for omphalocele immediately after birth and mechanical ventilation for several months. At present, she is 5.5 months of age, and still requires intensive care including oxygen administration and tube feeding. Patient 2 died at four days of age due to massive intracranial hemorrhage, while receiving intensive care including mechanical ventilation. The mother of patient 1 had several non-specific clinical features such as short stature and obesity. The father of patient 1 and the parents of patient 2 were clinically normal.

**Table 1:** Clinical features in patients 1 and 2.

	Patient 1	Patient 2	Upd(14)pat (n = 20)^c^
**Present age**	5.5 months	Deceased at 4 days	0–9 years
**Sex**	Female	Female	Male:Female = 9∶11
**Karyotype**	46,XX	46,XX	
**Pregnancy and delivery**			
Gestational age (weeks)	33	28	28–37
Delivery	Caesarean	Vaginal	Vaginal:Caesarean = 6:7
Polyhydramnios	Yes	No	20/20 (<28)^d^
Amnioreduction (weeks)	2× (28, 30)	No	6/6
Placentomegaly	Yes	No	10/10
**Growth pattern**			
Prenatal growth failure	No	No	1/13
Birth length (cm)	43 (WNR)^a^	34 (WNR)^a^	
Birth weight (kg)	2.84 (>90 centile)^a^	1.32 (WNR)^a^	
Postnatal growth failure	Yes	…	5/6
Present stature (cm)	56.3 (−3.0 SD)^b^	…	
Present weight (kg)	5.02 (−3.0 SD)^b^	…	
**Characteristic face**			
Frontal bossing	No	Yes	5/7
Hairy forehead	Yes	Yes	9/10
Blepharophimosis	Yes	No	14/15
Depressed nasal bridge	Yes	Yes	13/13
Anteverted nares	Yes	No	6/10
Small ears	Yes	Yes	11/12
Protruding philtrum	Yes	No	15/15
Puckered lips	No	No	3/10
Micrognathia	Yes	Yes	11/12
**Thoracic abnormality**			
Bell-shaped thorax	Yes	Yes	17/17
Mechanical ventilation	Yes	Yes	17/17
**Abdominal wall defect**			
Diastasis recti	…	…	15/17
Omphalocele	Yes	Yes	2/17^e^
**Others**			
Short webbed neck	Yes	Yes	14/14
Cardiac disease	No	Yes (PDA)	5/10
Inguinal hernia	No	No	2/6
Coxa valga	Yes	No	3/4
Joint contractures	Yes	No	8/10
Kyphoscoliosis	No	No	4/7
**Extra features**		Hydronephrosis	
		(bilateral)	

WNR: within the normal range; SD: standard deviation; and PDA: patent ductus arteriosus.

**a** Assessed by the gestational age- and sex-matched Japanese reference data from the Ministry of Health, Labor, and Welfare (http://www.e-stat.go.jp/SG1/estat/GL02020101.do).

**b** Assessed by the age- and sex-matched Japanese reference data..

**c** In the column summarizing the clinical features of 20 patients with upd(14)pat, the denominators indicate the number of cases examined for the presence or absence of each feature, and the numerators represent the number of cases assessed to be positive for that feature; thus, the differences between the denominators and the numerators denote the number of cases evaluated to be negative for that feature (adopted from reference [2]).

**d** Polyhydramnios has been identified by 28 weeks of gestation.

**e** Omphalocele is present in two cases with upd(14)pat and in two cases with epimutations [2].

### Sample preparation

We isolated genomic DNA (gDNA) and transcripts (*mRNAs*, *snoRNAs*, and *microRNAs*) from fresh leukocytes of patients 1 and the parents of patients 1 and 2, from fresh skin fibroblasts of patient 2, and from formalin-fixed and paraffin-embedded placental samples of patient 1 and similarly treated pituitary and adrenal samples of patient 2 (although multiple body tissues were available in patient 2, useful gDNA and transcript samples were not obtained from other tissues probably due to drastic post-mortem degradation). We also made metaphase spreads from leukocytes and skin fibroblasts. For comparison, we obtained control samples from fresh normal adult leukocytes, neonatal skin fibroblasts, and placenta at 38 weeks of gestation, and from fresh leukocytes of upd(14)pat/mat patients and formalin-fixed and paraffin-embedded placenta of a upd(14)pat patient [2], [3].

### Structural analysis of the imprinted region

**Figure 2:**
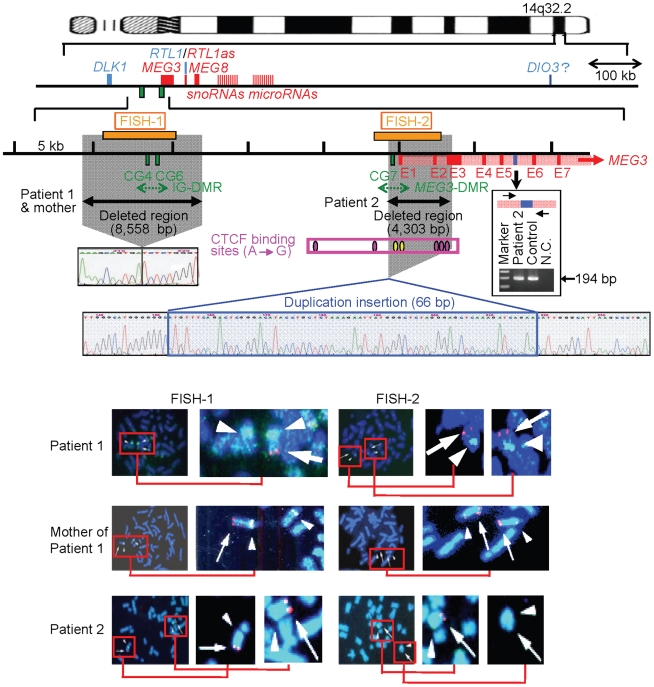
Physical map of the 14q32.2 imprinted region and the deleted segments in patient 1 and her mother and in patient 2 (shaded in gray). *PEGs* are shown in blue, *MEGs* in red, and the IG-DMR (CG4 and CG6) and the *MEG3*-DMR (CG7) in green. It remains to be clarified whether *DIO3* is a *PEG*, although mouse *Dio3* is known to be preferentially but not exclusively expressed from a paternally derived chromosome [35]. For *MEG3*, the isoform 2 with nine exons (red bars) and eight introns (light red segment) is shown (Ensembl; http://www.ensembl.org/index.html). Electrochromatograms represent the fusion point in patient 1 and her mother, and the fusion point accompanied by insertion of a 66 bp segment (highlighted in blue) with a sequence identical to that within *MEG3* intron 5 (the blue bar) in patient 2. Since PCR amplification with primers flanking the 66 bp segment at *MEG3* intron 5 has produced a 194 bp single band in patient 2 as well as in a control subject (shown in the box), this indicates that the 66 bp segment at the fusion point is caused by a duplicated insertion rather than by a transfer from intron 5 to the fusion point (if the 66 bp is transferred from the original position, a 128 bp band as well as a 194 bp band should be present in patient 2) (the marker size: 100, 200, and 300 bp). In the FISH images, the red signals (arrows) have been identified by the FISH-1 probe and the FISH-2 probe, and the light green signals (arrowheads) by the RP11-566I2 probe for 14q12 used as an internal control. The faint signal detected by the FISH-2 probe in patient 2 is consistent with the preservation of a ∼1.2 kb region identified by the centromeric portion of the FISH-2 probe.

We first examined the structure of the 14q32.2 imprinted region (Figure 2). Upd(14) was excluded in patients 1 and 2 as well as in the mother of patient 1 by microsatellite analysis (Table S1), and FISH analysis for the two DMRs identified a familial heterozygous deletion encompassing the IG-DMR alone in patient 1 and her mother and a *de novo* heterozygous deletion encompassing the *MEG3*-DMR alone in patient 2 (Figure 2). The microdeletions were further localized by SNP genotyping for 70 loci (Table S1) and quantitative real-time PCR (q-PCR) analysis for four regions around the DMRs (Figure S1A), and serial direct sequencing for the long PCR products harboring the deletion junctions successfully identified the fusion points of the microdeletions in patient 1 and her mother and in patient 2 (Figure 2). According to the NT_026437 sequence data at the NCBI Database (Genome Build 36.3) (http://preview.ncbi.nlm.nih.gov/guide/), the deletion size was 8,558 bp (82,270,449–82,279,006 bp) for the microdeletion in patient 1 and her mother, and 4,303 bp (82,290,978–82,295,280 bp) for the microdeletion in patient 2. The microdeletion in patient 2 also involved the 5′ part of *MEG3* and five of the seven putative CTCF binding sites A–G [10], and was accompanied by insertion of a 66 bp sequence duplicated from *MEG3* intron 5 (82,299,727–82,299,792 bp on NT_026437). Direct sequencing of the exonic or transcribed regions detected no mutation in *DLK1*, *MEG3*, and *RTL1*, although several cDNA polymorphisms (cSNPs) were identified (Table S1). Oligoarray comparative genomic hybridization identified no other discernible structural abnormality (Figure S1B).

### Methylation analysis of the two DMRs and the seven putative CTCF binding sites

**Figure 3:**
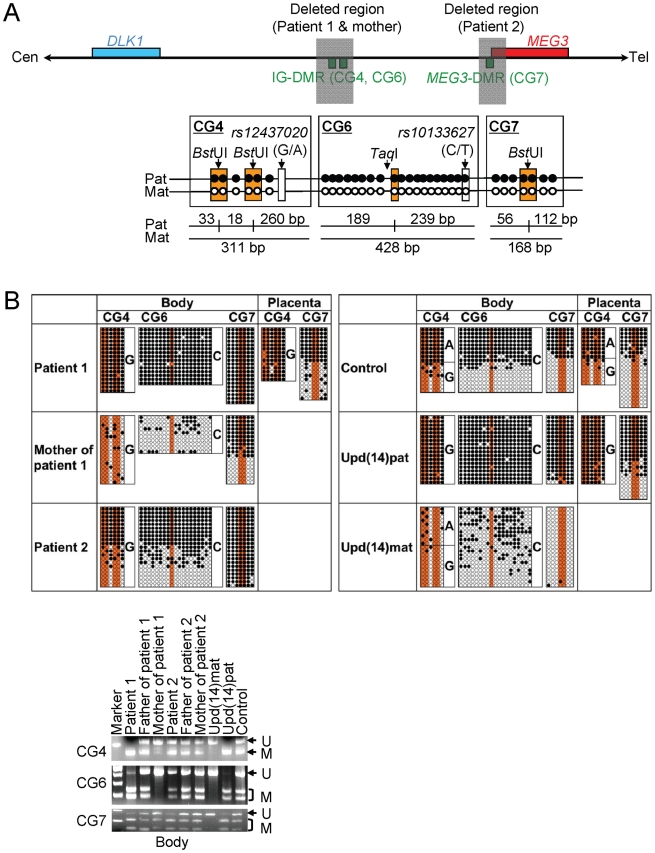
Methylation analysis of the IG-DMR (CG4 and CG6) and the *MEG3*-DMR (CG7). Filled and open circles indicate methylated and unmethylated cytosines at the CpG dinucleotides, respectively. (A) Structure of CG4, CG6, and CG7. Pat: paternally derived chromosome; and Mat: maternally derived chromosome. The PCR products for CG4 (311 bp) harbor 6 CpG dinucleotides and a G/A SNP (*rs12437020*), and are digested with *Bst*UI into three fragment (33 bp, 18 bp, and 260 bp) when the cytosines at the first and the second CpG dinucleotides and the fourth and the fifth CpG dinucleotides (indicated with orange rectangles) are methylated. The PCR products for CG6 (428 bp) carry 19 CpG dinucleotides and a C/T SNP (*rs10133627*), and are digested with *Taq*I into two fragment (189 bp and 239 bp) when the cytosine at the 9th CpG dinucleotide (indicated with an orange rectangle) is methylated. The PCR products for CG7 harbor 7 CpG dinucleotides, and are digested with *Bst*UI into two fragment (56 bp and 112 bp) when the cytosines at the fourth and the fifth CpG dinucleotides (indicated with orange rectangles) are methylated. These enzymes have been utilized for combined bisulfite restriction analysis (COBRA). (B) Methylation analysis. Upper part shows bisulfite sequencing data. The SNP typing data are also denoted for CG4 and CG6. The circles highlighted in orange correspond to those shown in Figure 3A. The relatively long CG6 was not amplified from the formalin-fixed and paraffin-embedded placental samples, probably because of the degradation of genomic DNA. Note that CG4 is differentially methylated in a control placenta and is massively hypermethylated in a upd(14)pat placenta, whereas CG7 is rather hypomethylated in a upd(14)pat placenta as well as in a control placenta. Lower part shows COBRA data. U: unmethylated clone specific bands (311 bp for CG4, 428 bp for CG6, and 168 bp for CG7); and M: methylated clone specific bands (260 bp for CG4, 239 bp and 189 bp for CG6, and 112 bp and 56 bp for CG7). The results reproduce the bisulfite sequencing data, and delineate normal findings of the father of patient 1 and the parents of patient 2.

We next studied methylation patterns of the previously reported IG-DMR (CG4 and CG6) and *MEG3*-DMR (CG7) (Figure 3A) [2], using bisulfite treated gDNA samples. Bisulfite sequencing and combined bisulfite restriction analysis using body samples revealed a hypermethylated IG-DMR and *MEG3*-DMR in patient 1, a hypomethylated IG-DMR and differentially methylated *MEG3*-DMR in the mother of patient 1, and a differentially methylated IG-DMR and hypermethylated *MEG3*-DMR in patient 2, and bisulfite sequencing using placental samples showed a hypermethylated IG-DMR and rather hypomethylated *MEG3*-DMR in patient 1 (Figure 3B).

**Figure 4:**
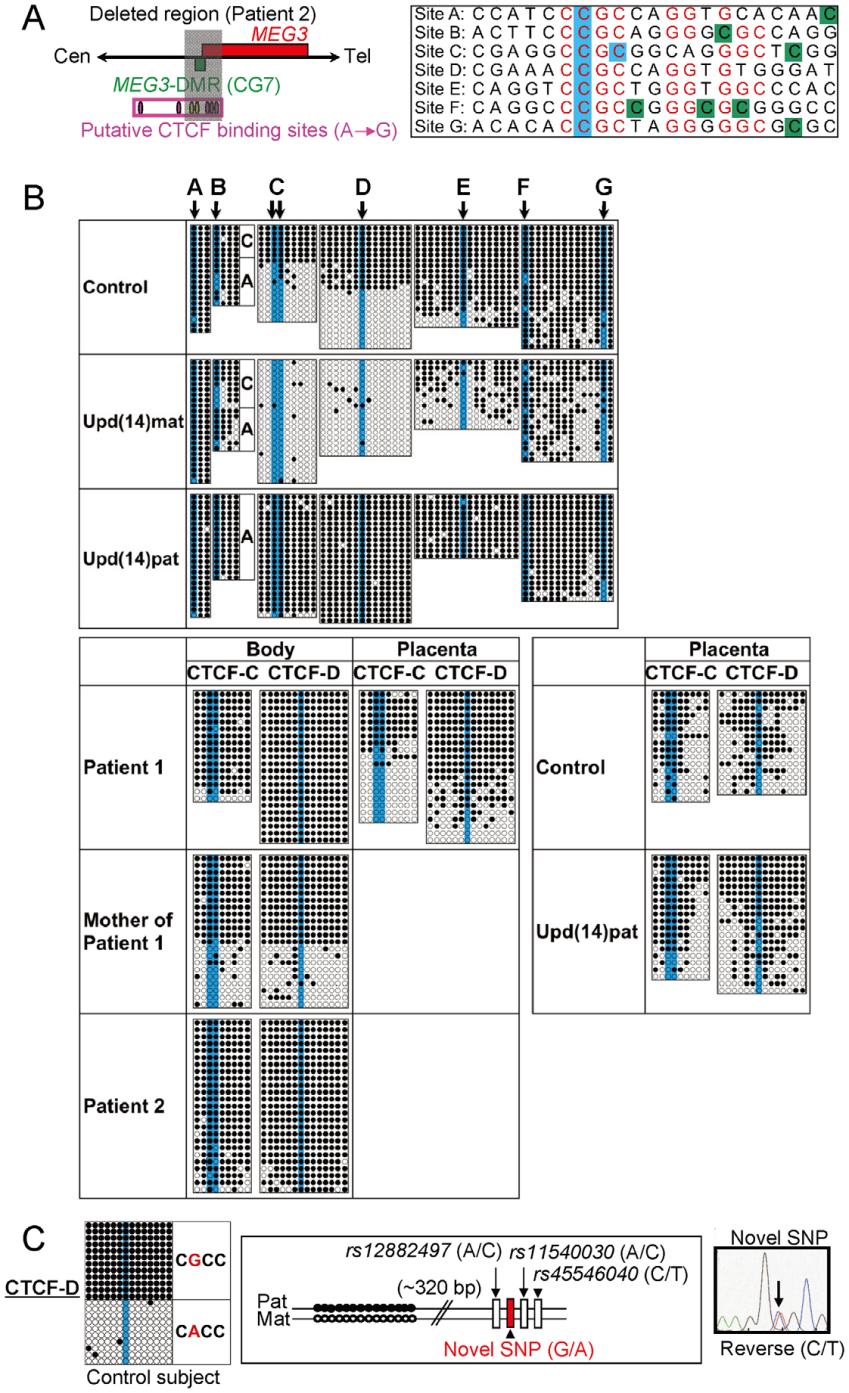
Methylation analysis of the putative CTCF protein binding sites A–G. (A) Location and sequence of the putative CTCF binding sites. In the left part, the sites C and D are painted in yellow and the remaining sites in purple. In the right part, the consensus CTCF binding motifs are shown in red letters; the cytosine residues at the CpG dinucleotides within the CTCF binding motifs are highlighted in blue, and those outside the CTCF binding motifs are highlighted in green [10]. (B) Methylation analysis. Upper part shows bisulfite sequencing data, using leukocyte genomic DNA samples. Since PCR products for the site B contain a C/A SNP (*rs11627993*), genotyping data are also indicated. The circles highlighted in blue correspond to those shown in Figure 4A. The sites C and D exhibit clear DMRs. Lower part indicates the results of the sites C and D using leukocyte and/or placental genomic DNA samples. The findings are similar to those of CG7. (C) Allele-specific methylation pattern of the CTCF binding site D. A novel G/A SNP has been identified in a single control subject, as shown on a reverse chromatogram delineating a C/T SNP pattern, while the previously reported three SNPs were present in a homozygous condition. Methylated and unmethylated clones are associated with the “G” and the “A” alleles, respectively.

We also examined methylation patterns of the seven putative CTCF binding sites by bisulfite sequencing (Figure 4A). The sites C and D alone exhibited DMRs in the body and were rather hypomethylated in the placenta (Figure 4B), as observed in CG7. Furthermore, to identify an informative SNP(s) pattern for allele-specific bisulfite sequencing, we examined a 349 bp region encompassing the site C and a 356 bp region encompassing the site D as well as a 300 bp region spanning the previously reported three SNPs near the site D, in 120 control subjects, the cases with upd(14)pat/mat, and patients 1 and 2 and their parents. Consequently, an informative polymorphism was identified for a novel G/A SNP near the site D in only a single control subject, and the parent-of-origin specific methylation pattern was confirmed (Figure 4C). No informative SNP was found in the examined region around the site C, and no other informative SNP was identified in the two examined regions around the site D, with the previously known three SNPs being present in a homozygous condition in all the subjects analyzed.

### Expression analysis of the imprinted genes

**Figure 5:**
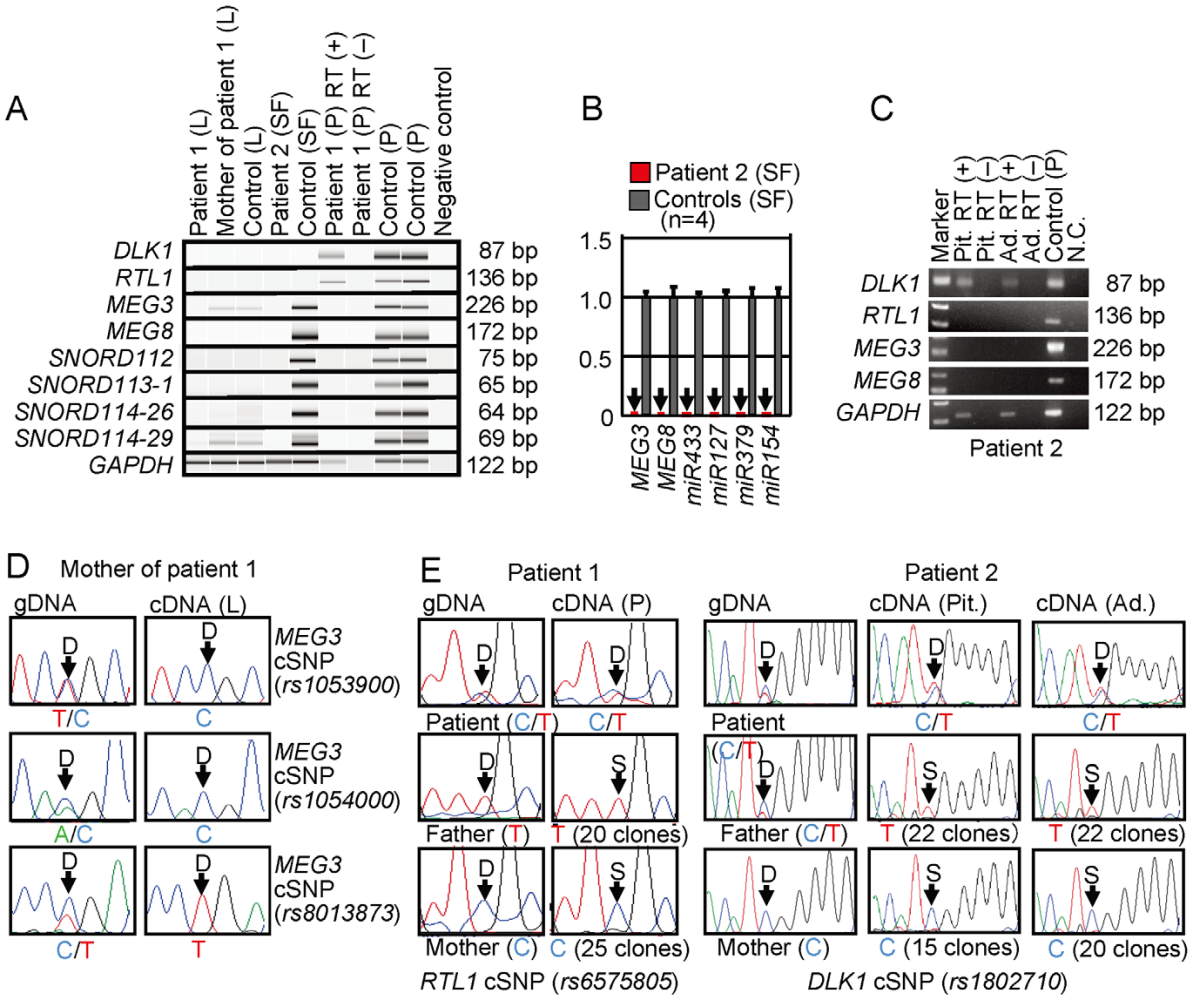
Expression analysis. (A) Reverse transcriptase (RT)-PCR analysis. L: leukocytes; SF: skin fibroblasts; and P: placenta. The relatively weak *GAPDH* expression for the formalin-fixed and paraffin-embedded placenta of patient 1 indicates considerable mRNA degradation. Since a single exon was amplified for *DLK1* and *RTL1*, PCR was performed with and without RT for the placenta of patient 1, to exclude the possibility of false positive results caused by genomic DNA contamination. (B) Quantitative real-time PCR (q-PCR) analysis of *MEG3*, *MEG8*, and *miRNAs,* using fresh skin fibroblasts (SF) of patient 2 and four control neonates. Of the examined *MEGs*, *miR433* and *miR127* are encoded by *RTL1as*. (C) RT-PCR analysis for the formalin-fixed and paraffin-embedded pituitary (Pit.) and the adrenal (Ad.) in patient 2. The bands for *DLK1* are detected in the presence of RT and undetected in the absence of RT, thereby excluding contamination of genomic DNA. (D) Monoallelic *MEG3* expression in the leukocytes of the mother of patient 1. The three cSNPs are present in a heterozygous status in gDNA and in a hemizygous status in cDNA. D: direct sequence. (E) Biparental *RTL1* expression in the placenta of patient 1 and biparental *DLK1* expression in the pituitary and adrenal of patient 2. D: direct sequence; and S: subcloned sequence. In patient 1, genotyping of *RTL1* cSNP (*rs6575805*) using gDNA indicates maternal origin of the “C” allele and paternal origin of the “T” allele, and sequencing analysis using cDNA confirms expression of maternally as well as paternally derived *RTL1*. Similarly, in patient 2, genotyping of *DLK1* cSNP (*rs1802710*) using gDNA denotes maternal origin of the “C” allele and paternal origin of the “T” alleles, and sequencing analysis using cDNA confirms expression of maternally as well as paternally inherited *DLK1*.

Finally, we performed expression analyses, using standard reverse transcriptase (RT)-PCR and/or q-PCR analysis for multiple imprinted genes in this region (Figure 5A–5C). For leukocytes, weak expression was detected for *MEG3* and *SNORD114-29* in a control subject and the mother of patient 1 but not in patient 1. For skin fibroblasts, although all *MEGs* but no *PEGs* were expressed in control subjects, neither *MEGs* nor *PEGs* were expressed in patient 2. For placentas, although all imprinted genes were expressed in control subjects, *PEGs* only were expressed in patient 1. For the pituitary and adrenal of patient 2, *DLK1* expression alone was identified.

Expression pattern analyses using informative cSNPs revealed monoallelic *MEG3* expression in the leukocytes of the mother of patient 1 (Figure 5D), and biparental *RTL1* expression in the placenta of patient 1 (no informative cSNP was detected for *DLK1*) and biparental *DLK1* expression in the pituitary and adrenal of patient 2 (*RTL1* was not expressed in the pituitary and adrenal) (Figure 5E), as well as maternal *MEG3* expression in the control leukocytes and paternal *RTL1* expression in the control placentas (Figure S2). Although we also attempted q-PCR analysis, precise assessment was impossible for *MEG3* in the mother of patient 1 because of faint expression level in leukocytes and for *RTL1* in patient 1 and *DLK1* in patient 2 because of poor quality of mRNAs obtained from formalin-fixed and paraffin-embedded tissues.

## Discussion

**Figure 6:**
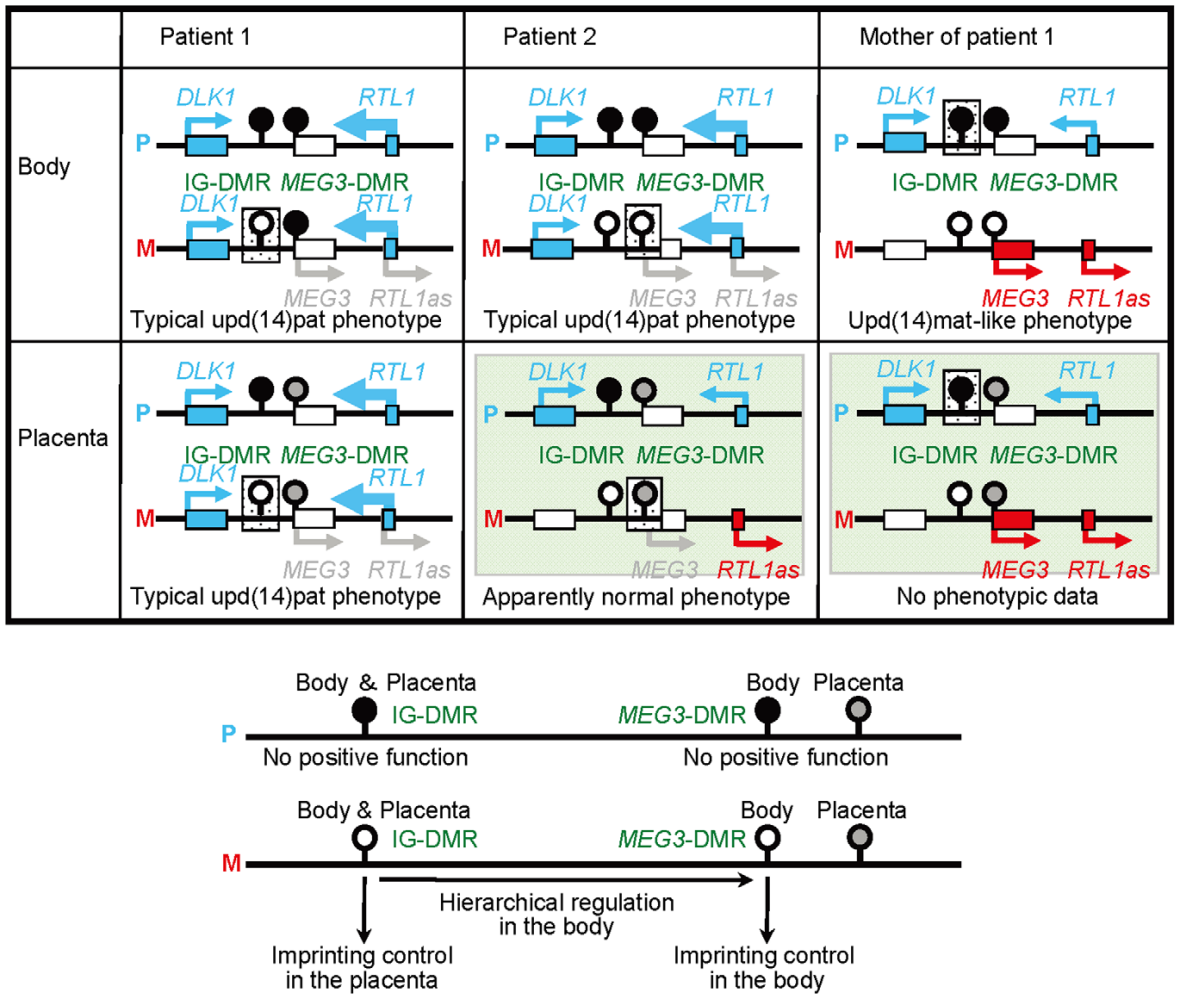
Schematic representation of the observed and predicted methylation and expression patterns. Deleted regions in patients 1 and 2 and the mother of patient 1 are indicated by stippled rectangles. P: paternally derived chromosome; and M: maternally derived chromosome. Representative imprinted genes are shown; these genes are known to be imprinted in the body and the placenta [2] (see also Figure S2). Placental samples have not been obtained in patient 2 and the mother of patient 1 (highlighted with light green backgrounds). Thick arrows for *RTL1* in patients 1 and 2 represent increased *RTL1* expression that is ascribed to loss of functional microRNA-containing *RTL1as* as a repressor for *RTL1* [26], [36]–[38]; this phenomenon has been indicated in placentas with upd(14)pat and in those with an epimutation and a microdeletion involving the two DMRs (Figure S3A and S3C) [2]. *MEG3* and *RTL1as* that are disrupted or predicted to have become silent on the maternally derived chromosome are written in gray. Filled and open circles represent hypermethylated and hypomethylated DMRs, respectively; since the *MEG3*-DMR is rather hypomethylated and regarded as non-DMR in the placenta [2] (see also Figure 3), it is painted in gray.

The data of the present study are summarized in Figure 6. Parental origin of the microdeletion positive chromosomes is based on the methylation patterns of the preserved DMRs in patients 1 and 2 and the mother of patient 1 as well as maternal transmission in patient 1. Loss of the hypomethylated IG-DMR of maternal origin in patient 1 was associated with epimutation (hypermethylation) of the *MEG3*-DMR in the body and caused paternalization of the imprinted region and typical upd(14)pat body and placental phenotypes, whereas loss of the hypomethylated *MEG3*-DMR of maternal origin in patient 2 permitted normal methylation pattern of the IG-DMR in the body and resulted in maternal to paternal epigenotypic alteration and typical upd(14)pat body, but no placental, phenotype. In this regard, while a 66 bp segment was inserted in patient 2, this segment contains no known regulatory sequence [11] or evolutionarily conserved element [12] (also examined with a VISTA program, http://genome.lbl.gov/vista/index.shtml). Similarly, while no control samples were available for pituitary and adrenal, the previous study in human subjects has shown paternal *DLK1* expression in adrenal as well as monoallelic *DLK1* and *MEG3* expressions in various tissues [11]. Furthermore, the present and the previous studies [2] indicate that this region is imprinted in the placenta as well as in the body. Thus, these results, in conjunction with the finding that the IG-DMR remains as a DMR and the *MEG3*-DMR exhibits a non-DMR in the placenta [2], imply the following: (1) the IG-DMR functions hierarchically as an upstream regulator for the methylation pattern of the *MEG3*-DMR on the maternally inherited chromosome in the body, but not in the placenta; (2) the hypomethylated *MEG3*-DMR functions as an essential imprinting regulator for both *PEGs* and *MEGs* in the body; and (3) in the placenta, the hypomethylated IG-DMR directly controls the imprinting pattern of both *PEGs* and *MEGs*. These notions also explain the epigenotypic alteration in the previous cases with epimutations or microdeletions affecting both DMRs (Figure S3).

It remains to be clarified how the IG-DMR and the *MEG3*-DMR interact hierarchically in the body. However, the present data, together with the previous findings in cases with epimutations [2], [5]–[8], imply that *MEG3*-DMR can remain hypomethylated only in the presence of a hypomethylated IG-DMR and is methylated when the IG-DMR is deleted or methylated irrespective of the parental origin. Furthermore, mouse studies have suggested that the methylation pattern of the postfertilization-derived *Gtl2-*DMR (the mouse homolog for the *MEG3*-DMR) is dependent on that of the germline-derive IG-DMR [13]. Thus, a preferential binding of some factor(s) to the unmethylated IG-DMR may cause a conformational alteration of the genomic structure, thereby protecting the methylation of the *MEG3*-DMR.

It also remains to be elucidated how the IG-DMR and the *MEG3*-DMR regulate the expression of both *PEGs* and *MEGs* in the placenta and the body, respectively. For the *MEG3*-DMR, however, the CTCF binding sites C and D may play a pivotal role in the imprinting regulation. The methylation analysis indicates that the two sites reside within the *MEG3*-DMR, and it is known that the CTCF protein with versatile functions preferentially binds to unmethylated target sequences including the sites C and D [10], [14]–[16]. In this regard, all the *MEGs* in this imprinted region can be transcribed together in the same orientation and show a strikingly similar tissue expressions pattern [1], [12], whereas *PEGs* are transcribed in different directions and are co-expressed with *MEGs* only in limited cell-types [1], [17]. It is possible, therefore, that preferential CTCF binding to the grossly unmethylated sites C and D activates all the *MEGs* as a large transcription unit and represses all the *PEGs* perhaps by influencing chromatin structure and histone modification independently of the effects of expressed *MEGs*. In support of this, CTCF protein acts as a transcriptional activator for *Gtl2* (the mouse homolog for *MEG3*) in the mouse [18].

Such an imprinting control model has not been proposed previously. It is different from the CTCF protein-mediated insulator model indicated for the *H19*-DMR and from the non-coding RNA-mediated model implicated for several imprinted regions including the KvDMR1 [19]. However, the KvDMR1 harbors two putative CTCF binding sites that may mediate non-coding RNA independent imprinting regulation [20], and the imprinting control center for Prader-Willi syndrome [21] also carries three CTCF binding sites (examined with a Search for CTCF DNA Binding Sites program, http://www.essex.ac.uk/bs/molonc/spa.html). Thus, while each imprinted region would be regulated by a different mechanism, a CTCF protein may be involved in the imprinting control of multiple regions, in various manners.

This imprinted region has also been studied in the mouse. Clinical and molecular findings in wildtype mice [1], [22], [23], mice with PatDi(12) (paternal disomy for chromosome 12 harboring this imprinted region) [13], [24], [25], and mice with targeted deletions for the IG-DMR (ΔIG-DMR) [22], [26] and for the *Gtl2-*DMR (Δ*Gtl2-*DMR) [27] are summarized in Table 2. These data, together with human data, provide several informative findings. First, in both the human and the mouse, the IG-DMR is differentially methylated in both the body and the placenta, whereas the *MEG3*/*Gtl2-*DMR is differentially methylated in the body and exhibits non-DMR in the placenta. Second, the IG-DMR and the *MEG3*/*Gtl2-*DMR show a hierarchical interaction on the maternally derived chromosome in both the human and the mouse bodies. Indeed, the *MEG3*/*Gtl2-*DMR is epimutated in patient 1 and mice with maternally inherited ΔIG-DMR, and the IG-DMR is normally methylated in patient 2 and mice with maternally inherited Δ*Gtl2-*DMR. Third, the function of the IG-DMR is comparable between human and mouse bodies and different between human and mouse placentas. Indeed, patient 1 has upd(14)pat body and placental phenotypes, whereas mice with the ΔIG-DMR of maternal origin have PatDi(12)-compatible body phenotype and apparently normal placental phenotype. It is likely that imprinting regulation in the mouse placenta is contributed by some mechanism(s) other than the methylation pattern of the IG-DMR, such as chromatin conformation [22], [28], [29].

**Table 2:** Clinical and molecular findings in wild-type and PatDi(12) mice and mice with maternally inherited ΔIG-DMR and Δ*Gtl2-*DMR.

	Wildtype	PatDi(12)	ΔIG-DMR (∼4.15 kb)^a^	Δ*Gtl2-*DMR (∼10 kb)^b^
				Neomycin cassette (+)
**<Body>**				
**Phenotype**	Normal	Abnormal^c^	PatDi(12) phenotype^c^	Normal at birth
				Lethal by 4 weeks
**Methylation pattern**				
IG-DMR	Differential	Methylated	Methylated^d^	Differential
*Gtl2*-DMR	Differential	Methylated	Epimutated^e^	Methylated^d^
**Expression pattern**				
*Pegs*	Monoallelic	Increased (∼2x)	Biparental	Grossly normal
			Increased (2x or 4.5x)^f^	
*Megs*	Monoallelic	Absent	Absent	Decreased (<0.2∼0.5x)^g^
**<Placenta>**				
**Phenotype**	Normal	Placentomegaly	Apparently normal	Not determined
**Methylation pattern**				
IG-DMR	Differential	Methylated	Not determined	Not determined
*Gtl2-DMR*	Non-DMR	Non-DMR	Not determined	Not determined
**Expression pattern**				
*Pegs*	Monoallelic	Not determined	Increased (1.5∼1.8x)^g^	Decreased (0.5∼0.85x)^g^
*Megs*	Monoallelic	Not determined	Decreased (0.6∼0.8x)^g^	Decreased (<0.1∼1.0)^g^
**Remark**			Paternal transmission^h^	Paternal transmission^i^
				Biparental transmission^j^

**a** The deletion size is smaller than that of patient 1 and her mother in this study, especially at the centromeric region.

**b** The microdeletion also involves *Gtl2*, and the deletion size is larger than that of patient 2 in this study.

**c** Body phenotype includes bell-shaped thorax with rib anomalies, distended abdomen, and short and broad neck.

**d** Hemizygosity for the methylated DMR of paternal origin.

**e** Hypermethylation of the maternally derived DMR.

**f** 2x *Dlk1* and *Dio3* expression levels and 4.5x *Rtl1* expression level. The markedly elevated *Rtl1* expression level is ascribed to a synergic effect between activation of the usually silent *Rtl1* of maternal origin and loss of functional microRNA-containing *Rtl1as* as a repressor for *Rtl1* [26], [36]–[38].

**g** The expression level is variable among examined tissues and examined genes.

**h** The ΔIG-DMR of paternal origin has permitted normal *Gtl2*-DMR methylation pattern, intact imprinting status, and normal phenotype in the body (no data on the placenta).

**i** The Δ*Gtl2*-DMR of paternal origin is accompanied by normal methylation pattern of the IG-DMR and variably reduced *Pegs* expression and increased *Megs* expression in the body, and has yielded severe growth retardation accompanied by perinatal lethality.

**j** The homozygous mutants have survived and developed into fertile adults, despite rather altered expression patterns of the imprinted genes.

Unfortunately, however, the data of Δ*Gtl2-*DMR mice appears to be drastically complicated by the retained neomycin cassette in the upstream region of *Gtl2*. Indeed, it has been shown that the insertion of a *lacZ* gene or a neomycin gene in the similar upstream region of *Gtl2* causes severely dysregulated expression patterns and abnormal phenotypes after both paternal and maternal transmissions [30], [31], and that deletion of the inserted neomycin gene results in apparently normal expression patterns and phenotypes after both paternal and maternal transmissions [31]. (In this regard, although a possible influence of the inserted 66 bp segment can not be excluded formally in patient 2, phenotype and expression data in patient 2 are compatible with simple paternalization of the imprinted region.) In addition, since the apparently normal phenotype in mice homozygous for Δ*Gtl2-*DMR is reminiscent of that in sheep homozygous for the callipyge mutation [32], a complicated mechanism(s) such as the polar overdominance may be operating in the Δ*Gtl2-*DMR mice [33]. Thus, it remains to be clarified whether the *MEG3*/*Gtl2-*DMR has a similar or different function between the human and the mouse.

Two points should be made in reference to the present study. First, the proposed functions of the two DMRs are based on the results of single patients. This must be kept in mind, because there might be a hidden patient-specific abnormality or event that might explain the results. For example, the abnormal placental phenotype in patient 1 might be caused by some co-incidental aberration, and the apparently normal placenta in patient 2 might be due to mosaicism with grossly preserved *MEG3*-DMR in the placenta and grossly deleted *MEG3*-DMR in the body. Second, the clinical features in the mother of patient 1 such as short stature and obesity are often observed in cases with upd(14)mat (Table S2). However, the clinical features are non-specific and appear to be irrelevant to the microdeletion involving the IG-DMR, because loss of the paternally derived IG-DMR does not affect the imprinted status [2], [26]. Indeed, *MEG3* in the mother of patient 1 showed normal monoallelic expression in the presence of the differentially methylated *MEG3*-DMR. Nevertheless, since the upd(14)mat phenotype is primarily ascribed to loss of functional *DLK1* (Figure S3B) [2], [34], it might be possible that the microdeletion involving the IG-DMR has affected a *cis*-acting regulatory element for *DLK1* expression (for details, see Note in the legend for Table S2). Further studies in cases with similar microdeletions will permit clarification of these two points.

In summary, the results show a hierarchical interaction and distinct functional properties of the IG-DMR and the *MEG3*-DMR in imprinting control. Thus, this study provides significant advance in the clarification of mechanisms involved in the imprinting regulation at the 14q32.2 imprinted region and the development of upd(14) phenotype.

## Materials and Methods

### Ethics statement

This study was approved by the Institutional Review Board Committees at National Center for Child health and Development, University College Dublin, and Dokkyo University School of Medicine, and performed after obtaining written informed consent.

### Primers

All the primers utilized in this study are summarized in Table S3.

### Sample preparation

For leukocytes and skin fibroblasts, genomic DNA (gDNA) samples were extracted with FlexiGene DNA Kit (Qiagen), and RNA samples were prepared with RNeasy Plus Mini (Qiagen) for *DLK1, MEG3, RTL1*, *MEG8* and *snoRNAs*, and with mirVana miRNA Isolation Kit (Ambion) for *microRNAs*. For paraffin-embedded tissues including the placenta, brain, lung, heart, liver, spleen, kidney, bladder, and small intestine, gDNA and RNA samples were extracted with RecoverAll Total Nucleic Acids Isolation Kit (Ambion) using slices of 40 µm thick. For fresh control placental samples, gDNA and RNA were extracted using ISOGEN (Nippon Gene). After treating total RNA samples with DNase, cDNA samples for *DLK1, MEG3, MEG8*, and *snoRNAs* were prepared with oligo(dT) primers from 1 µg of RNA using Superscript III Reverse Transcriptase (Invitrogen), and those for *microRNAs* were synthesized from 300 ng of RNA using TaqMan MicroRNA Reverse Transcription Kit (Applied Biosystems). For *RTL1*, cDNA samples were synthesized with *RTL1*-specific primers that do not amplify *RTL1as*. Control gDNA and cDNA samples were extracted from adult leukocytes and neonatal skin fibroblasts purchased from Takara Bio Inc. Japan, and from a fresh placenta of 38 weeks of gestation. Metaphase spreads were prepared from leukocytes and skin fibroblasts using colcemide (Invitrogen).

### Structural analysis

Microsatellite analysis and SNP genotyping were performed as described previously [2]. For FISH analysis, metaphase spreads were hybridized with a 5,104 bp FISH-1 probe and a 5,182 bp FISH-2 probe produced by long PCR, together with an RP11-566I2 probe for 14q12 used as an internal control [2]. The FISH-1 and FISH-2 probes were labeled with digoxigenin and detected by rhodamine anti-digoxigenin, and the RP11-566I2 probe was labeled with biotin and detected by avidin conjugated to fluorescein isothiocyanate. For quantitative real-time PCR analysis, the relative copy number to RNaseP (catalog No: 4316831, Applied Biosystems) was determined by the Taqman real-time PCR method using the probe-primer mix on an ABI PRISM 7000 (Applied Biosystems). To determine the breakpoints of microdeletions, sequence analysis was performed for long PCR products harboring the fusion points, using serial forward primers on the CEQ 8000 autosequencer (Beckman Coulter). Direct sequencing was also performed on the CEQ 8000 autosequencer. Oligoarray comparative genomic hybridization was performed with 1×244K Human Genome Array (catalog No: G4411B) (Agilent Technologies), according to the manufacturer's protocol.

### Methylation analysis

Methylation analysis was performed for gDNA treated with bisulfite using the EZ DNA Methylation Kit (Zymo Research). After PCR amplification using primer sets that hybridize both methylated and unmethylated clones because of lack of CpG dinucleotides within the primer sequences, the PCR products were digested with appropriate restriction enzymes for combined bisulfite restriction analysis. For bisulfite sequencing, the PCR products were subcloned with TOPO TA Cloning Kit (Invitrogen) and subjected to direct sequencing on the CEQ 8000 autosequencer.

### Expression analysis

Standard RT-PCR was performed for *DLK1*, *RTL1*, *MEG3*, *MEG8*, and *snoRNAs* using primers hybridizing to exonic or transcribed sequences, and one µl of PCR reaction solutions was loaded onto Gel-Dye Mix (Agilent). Taqman real-time PCR was carried out using the probe-primer mixtures (assay No: Hs00292028 for *MEG3* and Hs00419701 for *MEG8*; assay ID: 001028 for *miR433*, 000452 for *miR127*, 000568 for *miR379*, and 000477 for *miR154*) on the ABI PRISM 7000. Data were normalized against *GAPDH* (catalog No: 4326317E) for *MEG3* and *MEG8* and against *RNU48* (assay ID: 0010006) for the remaining *miRs*. The expression studies were performed three times for each sample.

To examine the imprinting status of *MEG3* in the leukocytes of the mother of patient 1, direct sequence data for informative cSNPs were compared between gDNA and cDNA. To analyze the imprinting status of *RTL1* in the placental sample of patient 1 and that of *DLK1* in the pituitary and adrenal samples of patient 2, RT-PCR products containing exonic cSNPs informative for the parental origin were subcloned with TOPO TA Cloning Kit, and multiple clones were subjected to direct sequencing on the CEQ 8000 autosequencer. Furthermore, *MEG3* expression pattern was examined using leukocyte gDNA and cDNA samples from multiple normal subjects and leukocyte gDNA samples from their mothers, and *RTL1* expression pattern was analyzed using gDNA and cDNA samples from multiple fresh normal placentas and leukocyte gDNA from the mothers.

## Supporting Information

Figure S1 Structural analysis. (A) Quantitative real-time PCR analysis (q-PCR) for four regions (q-PCR-1-4) in patient 2. The q-PCR-1 and q-PCR-2 regions are present in two copies whereas q-PCR-3 and q-PCR-4 regions are present in a single copy in patient 2. The four regions are present in two copies in the parents and a control subject, in a single copy in the two previously reported patients with microdeletions involving the examined regions (Deletion-1 and Deletion-2 are case 2 and case 3 in Kagami et al. [2], respectively), and in three copies in a hitherto unreported case with 46,XX,der(17)t(14;17)(q32.2;p13)pat who have three copies of the 14q32.2 imprinted region. Since the microsatellite locus *D14S985* is present in two copies (Table S1) and the *MEG3*-DMR is deleted (Figure 2) in patient 2, this has served to localize the breakpoints. (B) Oligoarray comparative genomic hybridization for a ∼1 Mb imprinted region. All the signals remain within the normal range (-1 SD ∼ +1 SD) (shaded in light blue) in patients 1 and 2.
(1.17 MB TIF)

Figure S2 Expression analysis. (A) Maternal *MEG3* expression in the leukocytes of normal subjects. Genotyping has been performed for three cSNPs using genomic DNA (gDNA) and cDNA of leukocytes from control subjects and gDNA samples of their mothers, indicating that both maternally and non-maternally (paternally) derived alleles are delineated in the gDNA, whereas maternally inherited alleles alone are identified in cDNA. These three cSNPs have also been studied in the mother of patient 1 (Figure 5D). (B) Paternal *RTL1* expression in the placenta of a normal subject. Genotyping has been carried out for *RTL1* cSNP using gDNA and cDNA samples of a fresh placenta and gDNA sample from the mother, showing that both maternally and non-maternally (paternally) derived alleles are delineated in the gDNA, whereas a non-maternally (paternally) inherited allele alone is detected in cDNA. This cSNP has also been examined in the placenta of patient 1 (Figure 5E). Furthermore, the results confirm that the primers utilized in this study have amplified *RTL1*, but not *RTL1as*.
(0.39 MB TIF)

Figure S3 Schematic representation of the observed and predicted methylation and expression patterns in previously reported cases with upd(14)pat/mat-like phenotypes and in normal and upd(14)pat/mat subjects. For the explanations of the illustrations, see the legend for Figure 6. Previous studies have indicated that (1) Epimutation-1, Deletion-1, Deletion-2, and Deletion-3 lead to maternal to paternal epigenotypic alteration; (2) Epimutation-2 results in paternal to maternal epigenotypic alteration; and (3) Deletion-4 and Deletion-5 have no effect on the epigenotypic status [2], . (A) Cases with typical or mild upd(14)pat phenotype. Epimutation-1: Hypermethylation of the IG-DMR and the *MEG3*-DMR of maternal origin in the body, and that of the IG-DMR of maternal origin in the placenta (the *MEG3*-DMR is rather hypomethylated in the placenta) (cases 6–8 in Kagami et al. [2]). Deletion-1: Microdeletion involving *DLK1*, the two DMRs, and *MEG3* on the maternally inherited chromosome (case 2 in Kagami et al. [2]). Deletion-2: Microdeletion involving *DLK1*, the two DMRs, *MEG3*, *RTL1*, and *RTL1as* on the maternally inherited chromosome (cases 3 and 5 in Kagami et al. [2]). Deletion-3: Microdeletion involving the two DMRs, *MEG3*, *RTL1*, and *RTL1as* on the maternally inherited chromosome (case 4 in Kagami et al. [2]). These findings are explained by the following notions: (1) Epimutation (hypermethylation) of the normally hypomethylated IG-DMR of maternal origin directly results in paternalization of the imprinted region in the placenta and indirectly leads to paternalization of the imprinted region in the body via epimutation (hypermethylation) of the usually hypomethylated *MEG3*-DMR of maternal origin. Thus, the epimutation (hypermethylation) is predicted to have impaired the IG-DMR as the primary target, followed by the epimutation (hypermethylation) of the *MEG3*-DMR after fertilization; (2) Loss of the hypomethylated *MEG3*-DMR of maternal origin leads to paternalization of the imprinted region in the body; and (3) Loss of the hypomethylated IG-DMR of maternal origin results in paternalization of the imprinted region in the placenta. Furthermore, epigenotype-phenotype correlations imply that the severity of upd(14)pat phenotype is primarily determined by the *RTL1* expression dosage rather than the *DLK1* expression dosage [2]. (B) Cases with upd(14)mat-like phenotype. Epimutation-2: Hypomethylation of the IG-DMR and the *MEG3*-DMR of paternal origin (Temple et al. [5], Buiting et al. [6], Hosoki et al. [7], and Zechner et al. [8]). Deletion-4: Microdeletion involving *DLK1*, the two DMRs, and *MEG3* on the paternally inherited chromosome (cases 9 and 10 in Kagami et al. [2]). Deletion-5: Microdeletion involving *DLK1*, the two DMRs, *MEG3*, *RTL1*, and *RTL1as* on the paternally inherited chromosome (case 11 in Kagami et al. [2] and patient 3 in Buiting et al. [6]). These findings are consistent with the following notions: (1) Epimutation (hypomethylation) of the normally hypermethylated IG-DMR of paternal origin directly results in maternalization of the imprinted region in the placenta and indirectly leads to maternalization of the imprinted region in the body through epimutation (hypomethylation) of the usually hypermethylated *MEG3*-DMR of paternal origin. Thus, epimutation (hypomethylation) is predicted to have affected the IG-DMR as the primary target, followed by the epimutation (hypomethylation) of the *MEG3*-DMR after fertilization; and (2) Loss of the hypermethylated DMRs of paternal origin has no effect on the imprinting status [2], [26], so that upd(14)mat-like phenotype is primarily ascribed to the additive effects of loss of functional *DLK1* and *RTL1* from the paternally derived chromosome (the effects of loss of *DIO3* appears to be minor, if any [2], [35]). Although the *MEGs* expression dosage is predicted to be normal in Deletion-4 and Deletion-5 and doubled in Epimutation-2 as well as in upd(14)mat, it remains to be determined whether the difference in the *MEGs* expression dosage has major clinical effects or not. (C) Normal and upd(14)pat/mat subjects.
(2.72 MB TIF)

Table S1 The results of microsatellite and SNP analyses.
(0.19 MB DOC)

Table S2 Clinical features in the mother of patient 1.
(0.09 MB DOC)

Table S3 Primers utilized in the present study.
(0.14 MB DOC)
