# Supplementary figures and images for: The IG-DMR and the MEG3-DMR at Human Chromosome 14q32.2: Hierarchical Interaction and Distinct Functional Properties as Imprinting Control Centers
Source: PLoS Genet. 2010 Jun 17;6(6):e1000992. doi: 10.1371/journal.pgen.1000992 (PMC2887472; doi:10.1371/journal.pgen.1000992)

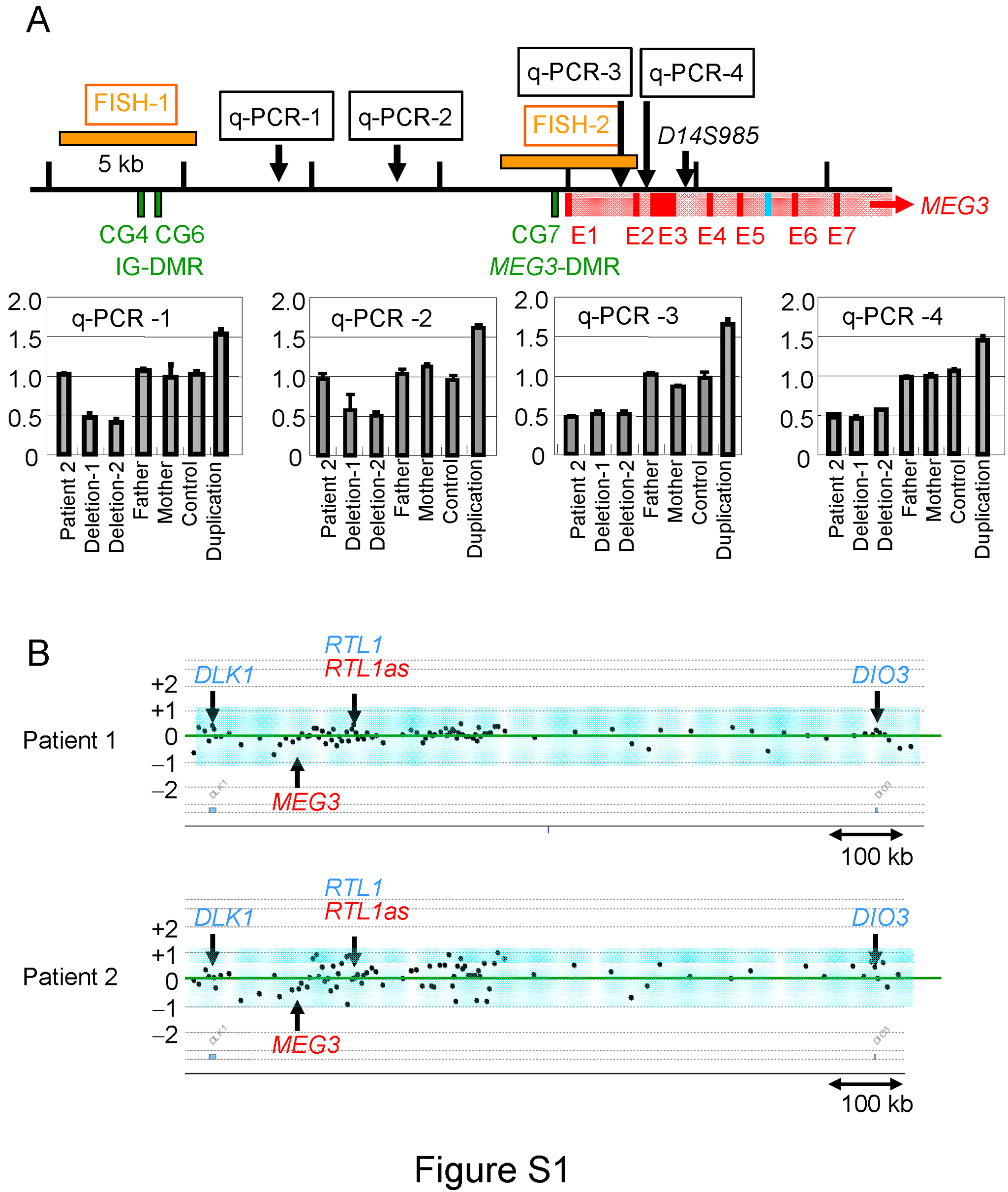

Supplement: Figure S1 — Structural analysis. (A) Quantitative real-time PCR analysis (q-PCR) for four regions (q-PCR-1-4) in patient 2. The q-PCR-1 and q-PCR-2 regions are present in two copies whereas q-PCR-3 and q-PCR-4 regions are present in a single copy in patient 2. The four regions are present in two copies in the parents and a control subject, in a single copy in the two previously reported patients with microdeletions involving the examined regions (Deletion-1 and Deletion-2 are case 2 and case 3 in Kagami et al. [2], respectively), and in three copies in a hitherto unreported case with 46,XX,der(17)t(14;17)(q32.2;p13)pat who have three copies of the 14q32.2 imprinted region. Since the microsatellite locus D14S985 is present in two copies (Table S1) and the MEG3-DMR is deleted (Figure 2) in patient 2, this has served to localize the breakpoints. (B) Oligoarray comparative genomic hybridization for a ∼1 Mb imprinted region. All the signals remain within the normal range (-1 SD ∼ +1 SD) (shaded in light blue) in patients 1 and 2. (1.17 MB TIF) [file pgen.1000992.s001.tif]

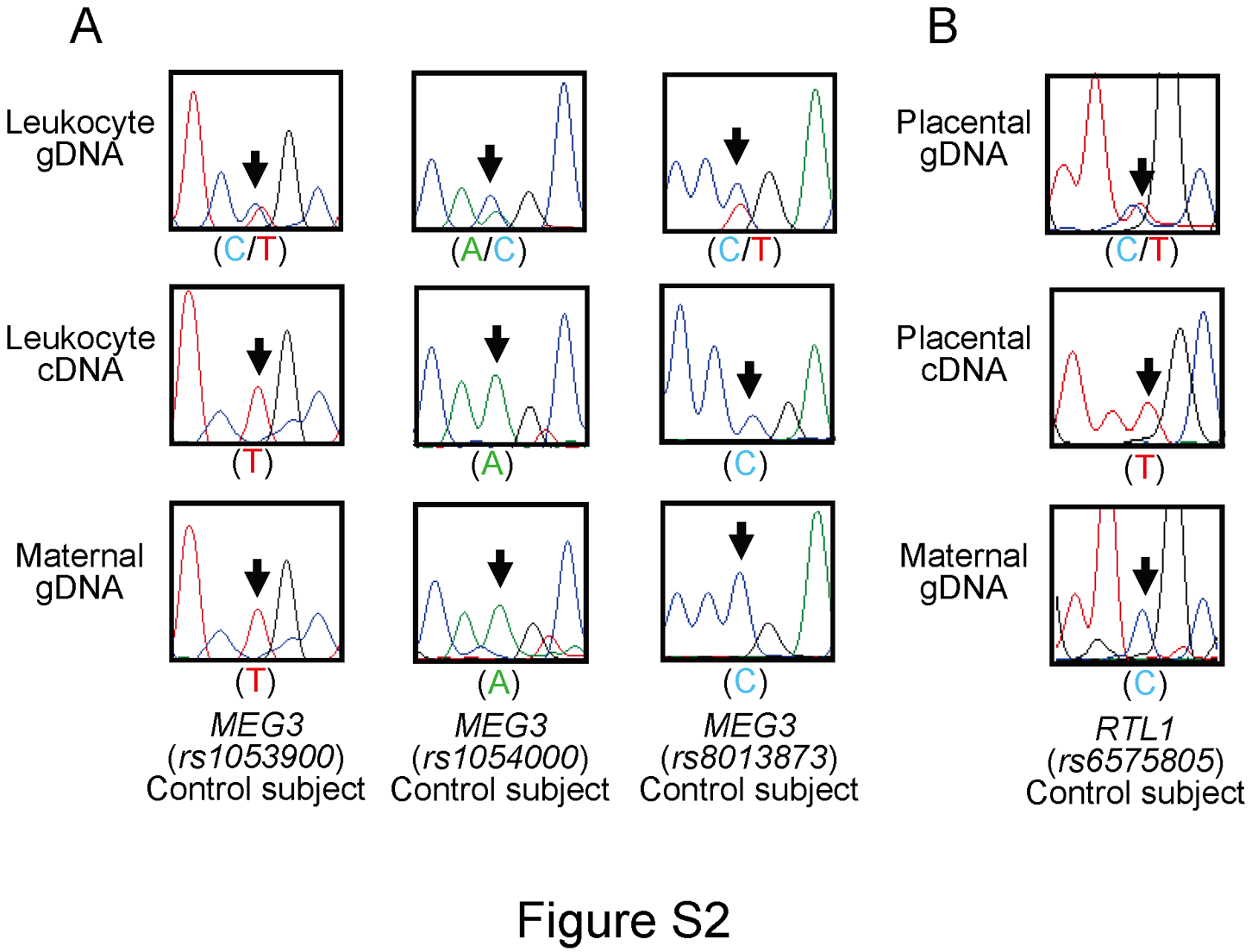

Supplement: Figure S2 — Expression analysis. (A) Maternal MEG3 expression in the leukocytes of normal subjects. Genotyping has been performed for three cSNPs using genomic DNA (gDNA) and cDNA of leukocytes from control subjects and gDNA samples of their mothers, indicating that both maternally and non-maternally (paternally) derived alleles are delineated in the gDNA, whereas maternally inherited alleles alone are identified in cDNA. These three cSNPs have also been studied in the mother of patient 1 (Figure 5D). (B) Paternal RTL1 expression in the placenta of a normal subject. Genotyping has been carried out for RTL1 cSNP using gDNA and cDNA samples of a fresh placenta and gDNA sample from the mother, showing that both maternally and non-maternally (paternally) derived alleles are delineated in the gDNA, whereas a non-maternally (paternally) inherited allele alone is detected in cDNA. This cSNP has also been examined in the placenta of patient 1 (Figure 5E). Furthermore, the results confirm that the primers utilized in this study have amplified RTL1, but not RTL1as. (0.39 MB TIF) [file pgen.1000992.s002.tif]

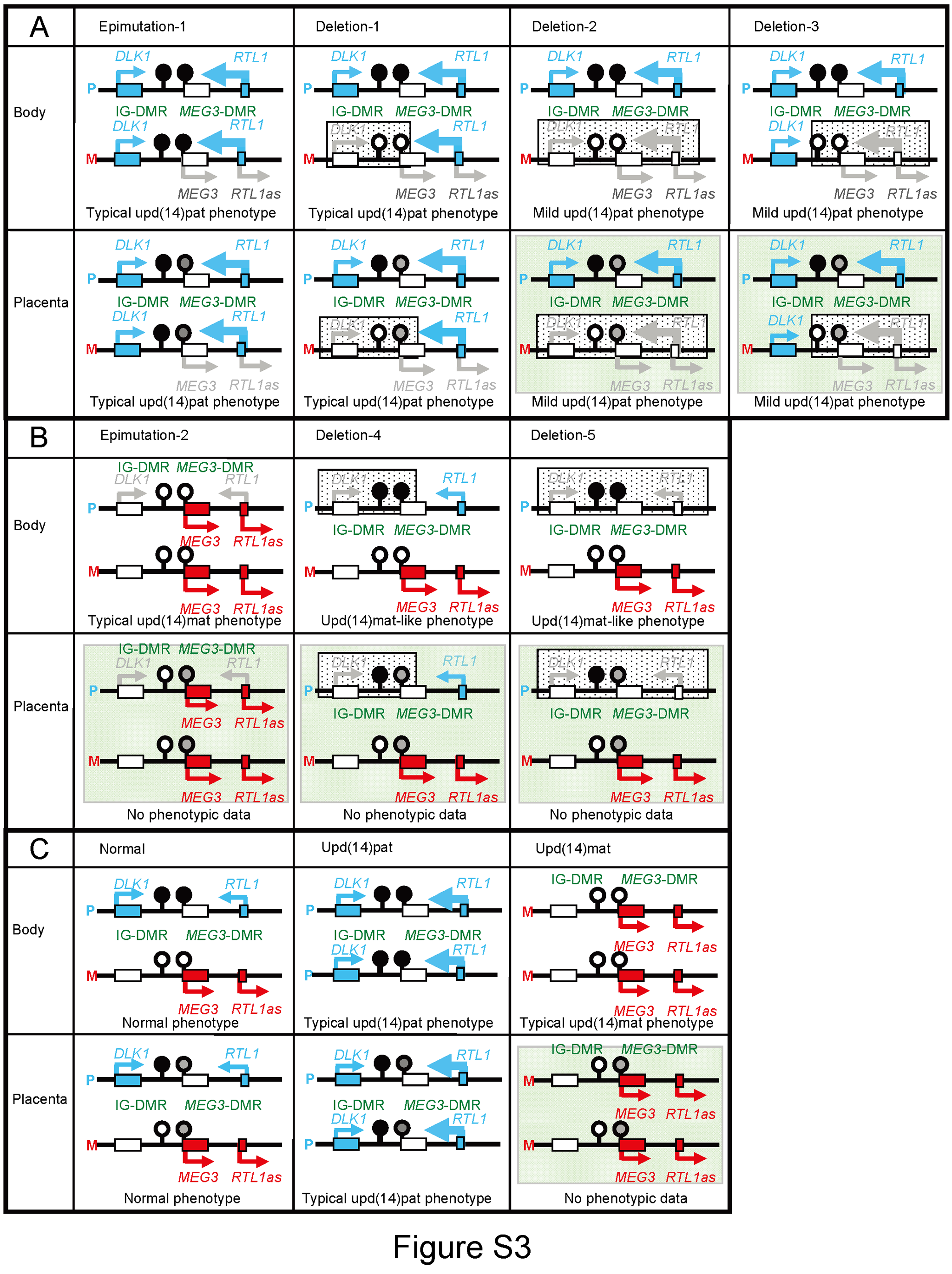

Supplement: Figure S3 — Schematic representation of the observed and predicted methylation and expression patterns in previously reported cases with upd(14)pat/mat-like phenotypes and in normal and upd(14)pat/mat subjects. For the explanations of the illustrations, see the legend for Figure 6. Previous studies have indicated that (1) Epimutation-1, Deletion-1, Deletion-2, and Deletion-3 lead to maternal to paternal epigenotypic alteration; (2) Epimutation-2 results in paternal to maternal epigenotypic alteration; and (3) Deletion-4 and Deletion-5 have no effect on the epigenotypic status [2], . (A) Cases with typical or mild upd(14)pat phenotype. Epimutation-1: Hypermethylation of the IG-DMR and the MEG3-DMR of maternal origin in the body, and that of the IG-DMR of maternal origin in the placenta (the MEG3-DMR is rather hypomethylated in the placenta) (cases 6–8 in Kagami et al. [2]). Deletion-1: Microdeletion involving DLK1, the two DMRs, and MEG3 on the maternally inherited chromosome (case 2 in Kagami et al. [2]). Deletion-2: Microdeletion involving DLK1, the two DMRs, MEG3, RTL1, and RTL1as on the maternally inherited chromosome (cases 3 and 5 in Kagami et al. [2]). Deletion-3: Microdeletion involving the two DMRs, MEG3, RTL1, and RTL1as on the maternally inherited chromosome (case 4 in Kagami et al. [2]). These findings are explained by the following notions: (1) Epimutation (hypermethylation) of the normally hypomethylated IG-DMR of maternal origin directly results in paternalization of the imprinted region in the placenta and indirectly leads to paternalization of the imprinted region in the body via epimutation (hypermethylation) of the usually hypomethylated MEG3-DMR of maternal origin. Thus, the epimutation (hypermethylation) is predicted to have impaired the IG-DMR as the primary target, followed by the epimutation (hypermethylation) of the MEG3-DMR after fertilization; (2) Loss of the hypomethylated MEG3-DMR of maternal origin leads to paternalization of the imprinted region in [file pgen.1000992.s003.tif]
