# Supplementary material for: The IG-DMR and the MEG3-DMR at Human Chromosome 14q32.2: Hierarchical Interaction and Distinct Functional Properties as Imprinting Control Centers
Source: PLoS Genet. 2010 Jun 17;6(6):e1000992. doi: 10.1371/journal.pgen.1000992 (PMC2887472; doi:10.1371/journal.pgen.1000992)
Supplement: Table S1 — The results of microsatellite and SNP analyses. (0.19 MB DOC) [file pgen.1000992.s004.doc]

**Table S1.** The Results of Microsatellite and SNP Analyses.

|  | |  |  | Patient 1 | Mother | Father |  | Patient 2 | Mother | Father |
| --- | --- | --- | --- | --- | --- | --- | --- | --- | --- | --- |
| **<Microsatellite analysis>** | | | | | | | | | | |
| Locus | Position | | Primer^a^ |  |  |  |  |  |  |  |
| *D14S250* | 14q32.2 | | D14S250F/R | 159 | 159 | 159 |  | 159/169 | 155/159 | 161/169 |
| *D14S1006* | 14q32.2 | | D14S1006F/R | 126/138 | 126/140 | 138/140 |  | 136/138 | 138/144 | 136/138 |
| *D14S985* | 14q32.2 | | D14S985F/R | 137 | 133/137 | 137 |  | 131/137 | 133/137 | 131/133 |
| *D14S1010* | 14q32.33 | | D14S1010F/R | 137/141 | 133/137 | 141/145 |  | 143/145 | 143/147 | 135/145 |
| *D14S292* | 14q32.33 | | D14S292F/R | 109/107 | 107/109 | 107 |  | 107/109 | 107/115 | 109/111 |
| *D14S1007* | 14q32.33 | | D14S1007F/R | 119 | 109/119 | 119/123 |  | 121/125 | 121 | 121/125 |
| **<SNP analysis>** | | | | | | | | | | |
| NCBI No. | NT_026437 (bp)^b^ | | Primer^a^ |  |  |  |  |  |  |  |
| *rs12435503* | 82191812 | | 1F/1R | C | C | C |  |  |  |  |
| *rs3759556* | 82192052 | | 1F/1R | A | A | A |  |  |  |  |
| *rs10139403* | 82194314 | | DLK1 99F/99R |  |  |  |  | A/G | A/G | G |
| *rs13329039* | 82194517 | | DLK1 2F/2R | A | A | A |  | A | A | A |
| *rs1135716* | 82195033 | | DLK1 2F/2R | C | C | C |  | C | C | C |
| *rs34686110* | 82195112 | | DLK1 3F/3R | A | A | A |  | A | A | A |
| *rs6575799* | 82198170 | | DLK1 4F/4R | G | G | G |  | G | G | G |
| *rs2273607* | 82198179 | | DLK1 4F/4R | G | G | G |  | G | G | G |
| *rs1058006* | 82198192 | | DLK1 4F/4R | A | A | A |  | A | A | A |
| *rs2273608* | 82198494 | | DLK1 21F/21R | C/T | C | C/T |  | C | C/T | C |
| *rs1757779* | 82199286 | | DLK1 100F/100R |  |  |  |  | C/T | C/T | T |
| *rs1802710* | 82200398 | | DLK1cSNP F/R | C | C | C |  | C/T | C | C/T |
| *rs34429112* | 82200437 | | DLK1 5F/5R | G | G | G |  | G | G | G |
| *rs2295660* | 82200533 | | DLK1 6F/6R | T | T | T |  | T | T | T |
| *rs1058009* | 82200613 | | DLK1 101F/101R | G | G | G |  | G | G | G |
| *rs35339877* | 82200674 | | DLK1 8F/8R | G | G | G |  | G | G | G |
| *rs12975* | 82200941 | | DLK1 8F/8R | C | C | C |  | C | C | C |
| *rs3198687* | 82200983 | | DLK1 8F/8R | C | C | C |  | C | C | C |
| *rs878110* | 82206242 | | 22F/22R | C/T | C | T |  |  |  |  |
| *rs11627672* | 82238816 | | 28F/28R | C | C | C |  |  |  |  |
| *rs10147396* | 82238817 | | 28F/28R | C/G | C | G |  |  |  |  |
| *rs10147577* | 82238840 | | 28F/28R | G | G | G |  |  |  |  |
| *rs10147404* | 82238853 | | 28F/28R | A/C | C | A |  |  |  |  |
| *rs10138933* | 82244838 | | 29F/29R | T | T | T |  |  |  |  |
| *rs10149782* | 82244962 | | 29F/29R | A | A | A |  |  |  |  |
| *rs8019641* | 82245096 | | 29F/29R | G | G | G |  |  |  |  |
| *rs2144820* | 82249282 | | 30F/30R | T | C/T | T |  |  |  |  |
| *rs2180388* | 82269346 | | 68F/68R | G | A/G | G |  |  |  |  |
| *rs12437020* | 82275703 | | CG4 F/R | G | G | G |  | G | G | G |
| *rs10133627* | 82277327 | | CG6 F/R | C | C | C |  | C | C | C |
| *rs1884538* | 82277410 | | 4F/4R | G | G | A/G |  | G | G | G |
| *rs1884539* | 82277539 | | 4F/4R | A | A | A |  | G | A/G | A/G |
| *rs12885923* | 82277562 | | 4F/4R | A | A | A |  | A | A | A |
| *rs12890188* | 82281176 | | 7F/7R | A/G | A | A/G |  | A | A/G | A/G |
| *rs12891580* | 82281189 | | 7F/7R | C/T | T | CT |  | T | C/T | C/T |
| *rs12889065* | 82287726 | | 10F/10R |  |  |  |  | A | A | A |
| *rs4906019* | 82287784 | | 10F/10R |  |  |  |  | C | C | C |
| *rs4906020* | 82289936 | | 14F/14R |  |  |  |  | A | A | A |
| *rs11627993* | 82290216 | | 9F/9R (CG9) |  |  |  |  | C | C | C |
| *rs12882497* | 82292169 | | MEG3 12F/12R |  |  |  |  | C | C | C |
| *rs12882497* | 82292169 | | CTCF-D SNP F/R | C | C | C |  | C | C | C |
| Novel SNP | 82292237 | | CTCF-D SNP F/R | G | G | G |  | G | G | G |
| *rs11540030* | 82292300 | | CTCF-D SNP F/R | C | C | C |  | C | C | C |
| *rs45546040* | 82292306 | | CTCF-D SNP F/R | C | C | C |  | C | C | C |
| *rs11540030* | 82292300 | | MEG3 isoform 2 1F/1R |  | C |  |  | C | C | C |
| *rs45546040* | 82292306 | | MEG3 isoform 2 1F/1R |  | C |  |  | C | C | C |
| *rs10134980* | 82293281 | | MEG3 13F/13R |  |  |  |  | C | A/C | C |
| *rs11624152* | 82294163 | | MEG3 16F/16R |  |  |  |  | G | G | G |
| *rs11540029* | 82294855 | | MEG3 18F/18R |  | C |  |  | C | C | C |
| *rs11540032* | 82294858 | | MEG3 18F/18R |  | T |  |  | T | T | T |
| *rs11540031* | 82295128 | | MEG3 isoform 2 3F/3R | C | C | C |  | C | C | C |
| *rs45518432* | 82295142 | | MEG3 isoform 2 3F/3R | T | T | T |  | T | T | T |
| *rs11540028* | 82295153 | | MEG3 isoform 2 3F/3R | C | C | C |  | C | C | C |
| *rs11540027* | 82295254 | | MEG3 isoform 2 3F/3R | A | A | A |  | A | A | A |
| *rs45497397* | 82295376 | | MEG3 isoform 2 3F/3R | T | T | T |  | T | T | T |
| *rs45470294* | 82295401 | | MEG3 isoform 2 3F/3R | T | T | T |  | T | T | T |
| *rs45497097* | 82295482 | | MEG3 isoform 2 3F/3R | G | G | G |  | G | G | G |
| *rs45617834* | 82295554 | | MEG3 isoform 2 3F/3R | C | C | C |  | C | C | C |
| *rs45617834* | 82295554 | | MEG3 isoform 2 3F/3R | C | C | C |  | C | C | C |
| *rs1053900* | 82301619 | | MEG3 cSNP(1F/1R) | C | C/T | C/T |  | C | C | C |
| *rs1054000* | 82301735 | | MEG3 cSNP(1F/1R) | A/C | A/C | A |  | C | C | C |
| *rs8013873* | 82301843 | | MEG3 cSNP(2F/2R) | C | C/T | T |  | C | C | C |
| *rs7158663* | 82319177 | | MEG3 88F/88R |  |  |  |  | G | A/G | A/G |
| *rs3742396* | 82319408 | | MEG3 88F/88R |  |  |  |  | A | A | A |
| *rs3742379* | 82319439 | | MEG3 88F/88R |  |  |  |  | C | C/G | C/G |
| *rs12884005* | 82347161 | | RTL1 8F/8R | G | G | G |  | G | G | G |
| *rs35695758* | 82347239 | | RTL1 7F/7R | G | G | G |  | G | G | G |
| *rs11623267* | 82348337 | | RTL1 4F/4R | C | C/G | C |  | C | C/G | C |
| *rs6575805* | 82348770 | | RTL1 cSNP F/R | T/C | C | T |  | C | C | T/C |
| *rs3825569* | 82350051 | | RTL1 cSNP (81F/81R) | C | C | C |  | C/T | C | T |

^a^ The primer sequences are shown in Table S3.

^b^ The NCBI database (Genome Build 36.3).

Loci involved in microdeletions in patient 1 and her mother and in patient 2 are shaded in gray; SNP genotyping data are not informative for the parental origin of the microdeletion positive chromosomes.

cSNPs are underlined.
