# Supplementary material for: The IG-DMR and the MEG3-DMR at Human Chromosome 14q32.2: Hierarchical Interaction and Distinct Functional Properties as Imprinting Control Centers
Source: PLoS Genet. 2010 Jun 17;6(6):e1000992. doi: 10.1371/journal.pgen.1000992 (PMC2887472; doi:10.1371/journal.pgen.1000992)
Supplement: Table S2 — Clinical features in the mother of patient 1. (0.09 MB DOC) [file pgen.1000992.s005.doc]

**Table S2.** Clinical Features in the Mother of Patient 1.

|  |  | The mother of patient 1 | Upd(14)mat (n=35)^h^ |
| --- | --- | --- | --- |
|  |  |  | Sporadic |
| Age | | 27 years | 0–30 years |
| Sex | | Female | Male:Female=17:18 |
| Karyotype | | 46,XX |  |
| Pregnancy and delivery | | | |
|  | Premature delivery | No | 10/25 |
|  | Gestational age (weeks) | 40 |  |
| Growth pattern | | | |
|  | Prenatal growth failure | No | 24/27 |
|  | Birth length (cm) | 48.0 (–0.7 SD)^a^ |  |
|  | Birth weight (kg) | 3.1 (–0.1 SD)^a^ |  |
|  | Postnatal growth failure | Yes | 26/32 |
|  | Present stature (cm) | 146 (–2.2 SD)^b^ |  |
|  | Present weight (kg) | 74.0 (+2.6 SD)^b^ |  |
| Pubertal development | | | |
|  | Early onset of puberty | No | 14/16 |
|  | Menarche (years) | 12.0 (–0.2 SD)^c^ |  |
| Others | | | |
|  | Mental retardation | No | 10/27 |
|  | Obesity (BMI) | Yes (35) | 14/34 |
|  | Hypotonia | Equivocal^d^ | 25/28 |
|  | Facial dysmorphism | Equivocal^e^ | 23/35 |
|  | Small hands | Yes | 24/27 |
|  | Scoliosis | No | 5/19 |
| Remarks | | Spontaneous abortions (3x)^f^ |  |
| Parental phenotype | | Short stature^g^ |  |

SD: standard deviation; BMI: body mass index.

^a^ Assessed by the gestational age- and sex-matched Japanese reference data from the Ministry of Health, Labor, and Welfare (<http://wwwdbtk.mhlw.go.jp/toukei/>).

^b^ Assessed by the age- and sex-matched Japanese reference data.

^c^ The menarchial age in Japanese girls is 12.25±1.25 years.

^d^ Allegedly, she had hypotonia during infancy.

^e^ She exhibits mild frontal bossing and shallow orbits.

^f^ Spontaneous abortions during the first trimester of the pregnancy; she also produced two normal boys.

^g^ The paternal height is 155 cm (–3.0 SD), and the maternal height is 146 cm (–2.2 SD).

^h^ In the column summarizing the clinical features of 35 cases with upd(14)mat, the denominators indicate the number of cases examined for the presence or absence of each feature, and the numerators represent the number of cases assessed to be positive for that feature; thus, the differences between the denominators and the numerators denote the number of cases evaluated to be negative for that feature (adopted from reference [2]).

**Note: Possible repression of *DLK1* by the microdeletion involving the IG-DMR**

The previous studies have indicated that upd(14)mat phenotype is primarily ascribed to loss of functional *DLK1* with an additional effect of loss of functional *RTL1* [2]. Thus, if the non-specific but upd(14)mat-like phenotype in the mother of patient 1 is related to the microdeletion on the paternally derived chromosome, the microdeletion might have affected a *cis*-acting regulatory element for the *DLK1* and/or *RTL1* expression. In this regard, although the data currently available argue against the possibility that the microdeletion have impaired the *RTL1* expression, it remains tenable that the microdeletion might have affected the *DLK1* expression.

1. If the microdeletion in the mother of patient 1 affects the *DLK1* and/or *RTL1* expression on the paternally derived chromosome, it is predicted that the microdeletion also impairs the *DLK1* and/or *RTL1* expression on the paternalized imprinted region of maternal origin in patient 1. In this regard, expression analysis using cSNP showed clear biparental *RTL1* expression in the placenta of patient 1 (Figure 5E). This argues against the possibility that the microdeletion affects the *RTL1* expression. Unfortunately, although such expression analysis using cSNP was also performed for *DLK1* in patient 1, no informative cSNP was identified by direct sequencing of the entire coding region. Furthermore, although quantitative real time PCR was also attempted for *DLK1*, this was virtually impossible because of small quantity and poor quality of mRNA obtained from formalin-fixed and paraffin-embedded placental samples.
2. Patient 1 manifests typical upd(14)pat phenotype that has primarily been ascribed to the markedly elevated *RTL1* expression rather than to the doubled *DLK1* expression [2] (the markedly elevated *RTL1* expression has been shown previously [2], and this phenomenon is explained by the synergic effect of two active copies of *RTL1* and the absence of functional microRNA-containing *RTL1as* as a repressor for *RTL1* [26,36-38]). Thus, if the microdeletion affects the function of *DLK1* on the paternalized imprinted region of maternal origin in patient 1, this would still be capable of causing typical upd(14)pat phenotype in patient 1, as observed in case 2 reported by Kagami et al. [2] (Deletion-1 in Figure S3A). However, if the microdeletion affects the function of *RTL1* on the paternalized imprinted region of maternal origin in patient 1, this would lead to relatively mild upd(14)pat phenotype in patient 1, as observed in case 3–5 described by Kagami et al. [2] (Deletion-2 and Deletion-3 in Figure S3A).
3. In the mice, the paternally derived *Dlk1* mutation has produced several upd(14)mat-like features such as pre- and postnatal growth deficiency, obesity, and facial abnormalities [34], whereas the paternally inherited *Rtl1* deletion has caused mild growth deficiency only [36]. Thus, the clinical features in the mother of patient 1 (Table S2) are more similar to those of *Dlk1* knockout mice than to those of *Rtl1* knockout mice.
4. In the mouse, the targeted deletion for the IG-DMR (ΔIG-DMR) of paternal origin has permitted normal *Gtl2*-DMR methylation pattern, imprinting status, and phenotype in the body [26]. In this regard, since the deleted region is larger in the mother of patient 1 than in the mouse with ΔIG-DMR (especially at the centromeric region), a possible regulatory element might reside in the non-DMR sequence that is deleted from patient 1 and her mother and is preserved in the ΔIG-DMR mouse.
