# Supplementary material for: The IG-DMR and the MEG3-DMR at Human Chromosome 14q32.2: Hierarchical Interaction and Distinct Functional Properties as Imprinting Control Centers
Source: PLoS Genet. 2010 Jun 17;6(6):e1000992. doi: 10.1371/journal.pgen.1000992 (PMC2887472; doi:10.1371/journal.pgen.1000992)
Supplement: Table S3 — Primers utilized in the present study. (0.14 MB DOC) [file pgen.1000992.s006.doc]

**Table S3.** Primers Utilized in the Present Study.

|  | Forward primer | Reverse primer | AT |
| --- | --- | --- | --- |
| <Microsatellite analysis> |  |  |  |
| D14S250 | GAAACTGGAACCACTGTGC | ACCCCTGCATTGTTTGAG | 55 |
| D14S1006 | TTCCACAGGGCAAGCAGTA | TTCTGGCAAAACCCAACC | 57 |
| D14S985 | CAGTGTGACCTTAAACAAGTCG | CCTGTGGGGTAGATACACGA | 57 |
| D14S1010 | AGATTCTGGACTTGCCAAC | GTAGTAGTCAGGGCTTCCTAGAG | 55 |
| D14S292 | CTGTGTGGTGCATCAATG | CATGAAGGCAGCCTCA | 55 |
| D14S1007 | AGCTCCTATATGTCTTCACACAG | CTCCATTCCCATACGTCC | 55 |
| <SNP and insertion polymorphism analysis> | | |  |
| 1F/1R | CCACCCAAAGATTGGGA | CACACATACCCAGCTGA | 60 |
| DLK1 99F/99R | CCGCTGTTAGGAGGACTTGA | TGGCACACAGTAGGCACTTC | 57 |
| DLK1 2F/2R | CCTGGTGGGGTGAATTGTATA | TTCTGCGTGGCCCCTACAA | 55 |
| DLK1 3F/3R | TCTTCATATGTCCCCACCTTT | ACGCAGAGCTGAGGTGAACA | 57 |
| DLK1 4F/4R | CTCGTGTATGGAGAGGAAGCT | CGCATGAAAAGCAGCATTCA | 57 |
| DLK1 21F/21R | GTCACCCCGCAGATGTTC | ACGCATCGGGACTTGAGA | 57 |
| DLK1 100F/100R | TCTTCAGACGGGGTCAGAGT | GGGAAGAAGGGGCAGTAAAC | 57 |
| DLK1cSNP F/R (rs1802710) | AACCCATGCGAGAACGAC | GCAGGTCTTGTCGATGAAGC | 57 |
| DLK1 5F/5R | CCCTGAGGGCGTTTACTATGT | TGCTGGCGGAGTTGGTCA | 57 |
| DLK1 6F/6R | CAACCCATGCGAGAACGA | GCACTTGTTGAGGAAGACGAT | 57 |
| DLK1 101F/101R | CCCGCTTTGACTTGTCTTGT | GGCGCCAAATTAATGACAAT | 57 |
| DLK1 8F/8R | GATCGACATGACCACCTTCA | CCTGGTTCTGTTGCCTTGTTTT | 57 |
| 22F/22R | CAGCAAAGCAGCCACATTT | TGGCTCCAGGGTTGTCTAGT | 57 |
| 28F/28R | AGGCTGACCCACGTATTCC | AAGCAGAGGCACACTCCAAG | 57 |
| 29F/29R | GCCTGGGGTACAGAGGAAGT | ACCGAGTGTGTCTGTGTGGA | 57 |
| 30F/30R | GAGGGACAGACAGGTTTCCA | GCCCACCTCCTTGAGTCTTT | 57 |
| 68F/68R | GCCTGAAAATGCTCTGGAAG | CTTCTGGGTCCAGCAAACTC | 57 |
| 4F/4R | CTCACAGTTGCCCATGGCT | GCCCTTCCCACTTCTGTCTC | 65 |
| 7F/7R | CTCGACTTGGCACAAAGG | TCCCTGGTGAGTGATTGG | 60 |
| 10F/10R | AATGCCTCTGTCCAGGAATG | CCAGCCTGGGTGACATAATAA | 57 |
| 14F/14R | GGCTCCTTAGGGACCCATCTT | CAAGGAAAAAGACCGTGGAA | 57 |
| 9F/9R | CAAACCCGTGGTGCTTTG | AAGGGCATGAGTTGACGTT | 60 |
| MEG3 12F/12R | TCCCTTCTTTGCTGCAATCT | TGGGTGGGGTTTATATGGAG | 57 |
| CTCF-C region F/R^a^ | gctcatcctcacctgctttc | gtggagatgcctgagctacc | 57 |
| CTCF-D region F/R^b^ | catgagttgtaagcggcaga | agggtgaattcaggcacaat | 57 |
| CTCF-D SNP F/R^c^ | gtgcggctagagcaatttgt | cgtcttccttttgcacatcc | 57 |
| MEG3 isoform 2 1F/1R | TCTGCGCCTCCATATAAACC | AGGATGGCCAACCACTCAC | 57 |
| MEG3 13F/13R | CTTTTGGTGAAATCGCCTTT | CCCTCCATCAGGAGAACAAA | 57 |
| MEG3 16F/16R | GTGGCCCACCTTCCTCTG | GAGGGAGGAGGGAGAAGAAA | 57 |
| MEG3 18F/18R | CCCTTTTGCTCTCTGCTGTT | TCTTCATCCTTTGCCATCCT | 57 |
| MEG3 isoform 2 3F/3R | CCTTTGATCAATTGCAGAGG | TTCCCCCAGAAAAGGATAGG | 57 |
| MEG3 cSNP(1F/1R) | ATCTGCAGGCTCTGCTTCTG | GCCAGGTGACCACAGGTATG | 57 |
| MEG3 cSNP(2F/2R) | GCCCTCCTGTGGTCTGAGTA | ACGATCACGAGGGGTCTCT | 57 |
| MEG3 88F/88R | GGATGCTGAGATTCGGGATA | TAGGAACACAACGGGACACA | 57 |
| RTL1 8F/8R | GTCAGAACCGCTACCTGGAG | GCTACCAAGGAATTCCAGGAC | 57 |
| RTL1 7F/7R | CAGCCATCCTCGTGCTACTG | CGCAAGACGACATCCTCATC | 57 |
| RTL1 4F/4R | CCAAAGGGGTGAAACTGAAC | GGAGATGTTGCGGGAGTAGA | 57 |
| RTL1 cSNP F/R (rs6575805) | CGTTTGGTTTGGAGCTTGA | TCCTGGCCATAAGAAAGCAC | 57 |
| RTL1 cSNP(81F/81R) | GGTGAACATGGCCTCTTCTG | AACGACCGTCTGAGAGTTGG | 57 |
| <Long PCR for FISH probe> |  |  |  |
| FISH probe 1 (IG-DMR) | ATACACCTCCAGGGATTCATGTGAGGAT | GAGGGTTGCCTAAGCATCAAGATTCCAT | 68 |
| FISH probe 2 (*MEG3*-DMR) | CTGGAAGACATTTGACTCGCCTGATGTA | AGATTCTGCCCGCCACGTTGGTTATGAA | 68 |
| <Quantitative real-time PCR >^d^ |  |  |  |
| q-PCR-1 | CATCTCGACTTGGCACAAAGG | AGGCAATGCACGGCAGAAA | 60 |
| q-PCR-2 | TCACCCTGCACTGCCATGT | CGCTGTCTGACGCTGAAAGG | 60 |
| q-PCR-3 | GGATTTCTGCTTTTCCCTGTAGCA | CCCCAGCCCCAGAGGAA | 60 |
| q-PCR-4 | GGCCTGCTGCCCATCTACAC | CGCCACGTTGGTTATGAAATG | 60 |

| <Long PCR for breakpoint determination> | | |  |
| --- | --- | --- | --- |
| Deletion analysis (patient 1 and her mother) | CAACAAATGAGAAAACAGCAGAGT | TCTTGAAAGTTTACATCCCCAAGT | 60 |
| Deletion analysis (patient 2) | GTGGCTAATAAACGTTCTCCTGTT | CACTTTCCACAGCAATTTACAAAG | 60 |
| <Direct sequence for breakpoint determination> | | |  |
| Patient 1 and her mother | TCTTGAAAGTTTACATCCCCAAGT |  | 55 |
| Patient 2 | GTGGCTAATAAACGTTCTCCTGTT |  | 55 |
| <MEG3 intron 5 including the inserted 66 bp sequence> | | |  |
| Patient 2 | CCATGCCCAGCCCAGCCCTATAGTA | GGGTGAGAAATGTCCAGAGC | 57 |
| <Direct sequence for mutation analysis> | | |  |
| DLK1 1F/1R | GGAGGCGGTACGAAAAGG | GTTGGGGCTCACGAGACG | 57 |
| DLK1 2F/2R | CCTGGTGGGGTGAATTGTATA | TTCTGCGTGGCCCCTACAA | 55 |
| DLK1 3F/3R | TCTTCATATGTCCCCACCTTT | ACGCAGAGCTGAGGTGAACA | 57 |
| DLK1 4F/4R | CTCGTGTATGGAGAGGAAGCT | CGCATGAAAAGCAGCATTCA | 57 |
| DLK1 5F/5R | CCCTGAGGGCGTTTACTATGT | TGCTGGCGGAGTTGGTCA | 57 |
| DLK1 6F/6R | CAACCCATGCGAGAACGA | GCACTTGTTGAGGAAGACGAT | 57 |
| DLK1 7F/7R | AACCCCTCTCCTCACCGA | GCGAACACCACAAAGATTAGG | 55 |
| DLK1 8F/8R | GATCGACATGACCACCTTCA | CCTGGTTCTGTTGCCTTGTTTT | 57 |
| MEG3 isoform 2 1F/1R | TCTGCGCCTCCATATAAACC | AGGATGGCCAACCACTCAC | 57 |
| MEG3 isoform 2 2F/2R | CTCCCATGCCATAGGGTCT | GGAGGGTTGAAGTACCGTGA | 55 |
| MEG3 isoform 2 3F/3R | CCTTTGATCAATTGCAGAGG | TTCCCCCAGAAAAGGATAGG | 57 |
| MEG3 isoform 2 4F/4R | GCCCGACCTCTCTGAAGAT | GGTCTCTGCAAAGCCCCTAC | 57 |
| MEG3 isoform 2 5F/5R | CAGGTCCCTGCTGTCATCTT | GTGTCCTTGTGTCCATGAGC | 57 |
| MEG3 isoform 2 6F/6R | CTGGCGGTGTTTTTCAGTTT | GGCTTAAGAAGCCACATCG | 57 |
| MEG3 isoform 2 7F/7R | TGTTGCCTTCTTCCTCGTCT | CTGTGGTGGTCGAGTCCTTT | 55 |
| MEG3 isoform 2 8F/8R | CTTCGGTGTGTTTGGCTTTT | TCCTGGGTCTCAAGAAAGCA | 57 |
| MEG3 isoform 2 9F/9R | CGGCCTGAGTGAGGTTCTAC | AGGCCACATGTGTGTTCTTT | 57 |
| MEG3 isoform 2 10F/10R | GCCTTCATCTGCAACTGTGT | AGCACATCCATCAGTTTTTGTT | 57 |
| RTL1 1F/1R | CCCCGAAGTCGACTGGAT | TCCAGCCTGCTGAAGCTC | 57 |
| RTL1 2F/2R | CGGATCCGATGATCTTTCTG | AAACGGCTGGTAGCTCTTCA | 57 |
| RTL1 3F/3R | ACCGAAGATGTGTGGAAAGC | TCGATGAAGTTTCGCAGAGA | 57 |
| RTL1 4F/4R | CCAAAGGGGTGAAACTGAAC | GGAGATGTTGCGGGAGTAGA | 57 |
| RTL1 5F/5R | ATCCAAATCGACGACCAAAC | CGGCAGTGTTCCTCTGGA | 57 |
| RTL1 6F/6R | CTGCCACCTGTGAGAAACCT | ATCCAGGATGAGTCGTAGGG | 57 |
| RTL1 7F/7R | CAGCCATCCTCGTGCTACTG | CGCAAGACGACATCCTCATC | 57 |
| RTL1 8F/8R | GTCAGAACCGCTACCTGGAG | GCTACCAAGGAATTCCAGGAC | 57 |
| <Methylation analysis> |  |  |  |
| CG4 F/R | TTTTATTATTGAATTGGGTTTGTTAGT | ACAATTCCTACTACAAAATTTCAACA | 57 |
| CG6 F/R | GTTAAGAGTTTGTGGATTTGTGAGAAATG | CTAAAAATCACCAAAACCCATAAAATCAC | 57 |
| CG7 F/R | TTGTGTTTGAATTTATTTTGTTT | CCCCAAATTCTATAACAAATTACT | 57 |
| CTCF-A F/R | AATGGTTTGAAAGAAAGGTTG | CAATACAAAATAAATACCACTCCA | 55 |
| CTCF-B F/R | GGAGAGTGGGGTTTATTGTGAA | AACCCTACAACCCCACAAAA | 60 |
| CTCF-C F/R | GAGGGTTTTTATTTGTTAGGGATT | TCCCCACACACATACCCTTT | 55 |
| CTCF-D F/R | GTTTATATTTGGGAATTAGTTATGT | TCAAACACAATATATAAAAAAAATC | 55 |
| CTCF-D allele-specific analysis^e^ | gtttatatttgggaattagttatgt | caacaacaaaacccaaaatcaa | 52 |
| CTCF-E F/R | GTTTGGAGGATTGGGTTTTT | CCAACCACCACCAACCAA | 50 |
| CTCF-F & G F/R | GTTTGAGATTTGTTGGGTATTT | CAACTCAAAACCCAAAATAAC | 55 |
| <RT-PCR analysis > |  |  |  |
| DLK1 cSNP F/R | AACCCATGCGAGAACGAC | GCAGGTCTTGTCGATGAAGC | 57 |
| MEG3 (exons 7–9, isoform 2) | TGCGGAAGAGGCCCTGAT | GTCCAGAGTCTCTGGGTCCA | 54 |
| RTL1 cSNP F/R^f^ | CGTTTGGTTTGGAGCTTGA | TCCTGGCCATAAGAAAGCAC | 57 |
| MEG8 (exons 2–3) | CAGTGTTGCCTGGGTCTGA | ATCCCCTTGAAAGAGCAGGA | 57 |
| GAPDH (exons 2–5, isoform 1) | Taqman probe-primer mixture (Catalog No. 4326317E) | | 60 |
| SNORD112 | TGGACCAATGATGAGACAGTG | TGGACCTCAGTGTTTTGTGC | 57 |
| SNORD113-1 | GAGTGATGAATAGTTCTGTGGCATA | GGACTTCAGAGTTTAGGGTTTAATCA | 57 |
| SNORD114-26 | GATGATGAGCACTGGTGGAG | GACCTCAGAGTTCCAGACACG | 57 |
| SNORD114-29 | TGGATCGATGATGACTACTGG | CAGACCTCAGAGTTCCAGACA | 57 |

| <RT-PCR direct sequencing> |  |  |  |
| --- | --- | --- | --- |
| DLK1 cSNP (rs1802710) | AACCCATGCGAGAACGAC | GCAGGTCTTGTCGATGAAGC | 57 |
| MEG3 cSNPs (rs1053900) | ATCTGCAGGCTCTGCTTCTG | GCCAGGTGACCACAGGTATG | 57 |
| MEG3 cSNPs (rs1054000 & rs8013873) | GCCCTCCTGTGGTCTGAGTA | ACGATCACGAGGGGTCTCT | 57 |
| RTL1 cSNP (rs6575805) | CGTTTGGTTTGGAGCTTGA | TCCTGGCCATAAGAAAGCAC | 57 |
| <RTL1-specific q PCR>^g^ |  |  |  |
| RTL1 / Adaptor | TGCCACTTACCAGACTTGCACAGCAAA  GAGGG | AGAGGTACCGGATCCGACTCGAGTCGA  CATCG | 70 |

^a^ The primers were utilized to search a possible SNP in a 349 bp region encompassing the CTCF binding site C.

^b^ The primers were utilized to search a possible SNP in a 356 bp region encompassing the CTCF binding site D.

^c^ The primers were utilized to amplify a 300 bp segment harboring three known SNPs in the vicinity of the CTCF binding site D; the novel SNP detected in a single control subject was identified with these primers.

^d^ Taqman probes used are: q-PCR-1, TAGAAGTCATTCAAACCC; q-PCR-2, CTGCCCTCTGTTTGATT; q-PCR-3, CCGCTGGGTGGCCCA; and q-PCR-4, TCACGGTACTTCAACCC.

^e^ The primers amplify a 705 bp segment including the CTCF binding site D and the novel SNP identified in a single control subject.

^f^ The cDNA synthesis for *RTL1* has been performed with *RTL1*-specifc primers that do not amplify *RTL1as*.

^g^ The cDNA synthesis was performed using the following primer with adaptor and oligo(dt): CTGATCTAGAGGTACCGGATCCGACTCGAGTCGACATCGTTTTTTTTTTTTTTTTTTT.

The probe-primer mixtures utilized for Taqman real-time q-PCR are: assay No: Hs00171584 for *DLK1*, Hs00292028 for *MEG3*, and Hs00419701 for *MEG8*; and assay ID: 001028 for *miR433*, 000452 for *miR127*, 000568 for *miR379*, and 000477 for *miR154*.
